# Targeted-Modified MultiTransm Microelectrode Arrays Simultaneously Track Dopamine and Cellular Electrophysiology in Nucleus Accumbens during Sleep–Wake Transitions

**DOI:** 10.34133/research.0944

**Published:** 2025-10-09

**Authors:** Qianli Jia, Zhaojie Xu, Yu Wang, Yiming Duan, Yu Liu, Jin Shan, Jiale Ma, Qi Li, Jinping Luo, Yan Luo, Ying Wang, Shumin Duan, Yanqin Yu, Mixia Wang, Xinxia Cai

**Affiliations:** ^1^ State Key Laboratory of Transducer Technology, Aerospace Information Research Institute, Chinese Academy of Sciences, Beijing 100190, China.; ^2^ University of Chinese Academy of Sciences, Beijing 100049, China.; ^3^ NHC and CAMS Key Laboratory of Medical Neurobiology, MOE Frontier Science Center for Brain Research and Brain-Machine Integration, School of Brain Science and Brain Medicine, Zhejiang University, Hangzhou, China.; ^4^ Department of Anesthesiology, Ruijin Hospital, Shanghai Jiaotong University School of Medicine, Shanghai 200025, China.

## Abstract

Cellular-level electrophysiological and neurotransmitter signals serve as key biomarkers of sleep depth, offering insights into the dynamic sleep transitions and the neural mechanisms underlying sleep regulation. Microelectrode arrays (MEAs) provide an innovative solution for in situ, simultaneous detection of these signals with high spatial and temporal resolution. However, despite substantial progress in electrode material development, current multimodal MEA systems remain fundamentally constrained by partial integration. This study aims to address the performance limitations of multimodal MEAs by developing a MultiTransm MEA (MT MEA), integrating a 3-electrode system with site-specific surface modifications: platinum nanoparticle (PtNP)/poly(3,4-ethylene dioxythiophene):poly(styrene sulfonate) (PEDOT:PSS)-modified sites for electrophysiology, PtNP/PEDOT:PSS/Nafion-modified sites for dopamine sensing, and iridium oxide (IrOx)-based on-probe reference electrodes. The directional surface modification strategy was employed to enable compact integration, minimize inter-channel crosstalk, preserve high spatiotemporal resolution for both electrophysiological and electrochemical detection, and ensure long-term operational stability. By incorporating electroencephalography (EEG) and electromyography (EMG), MT MEAs enable real-time in vivo monitoring of sleep dynamics within the nucleus accumbens. Three distinct spike types were identified, whose coordinated activity shaped the sleep architecture. In addition, EEG and local field potential (LFP) signals exhibited distinct patterns during wakefulness, indicating region-specific neural processing. Notably, dopamine release was lowest during non-rapid eye movement (NREM) sleep and peaked during wakefulness, suggesting a neuromodulatory role in sleep–wake transitions. These results demonstrate that MT MEAs are powerful tools for probing neural and neurochemical activity across sleep states, offering new insights into the physiological regulation of sleep.

## Introduction

Monitoring neural signals during sleep is crucial to understanding brain functionality and well-being. These signals offer valuable insights into the operational modes of the brain during rest, providing information regarding sleep phases [1], memory consolidation [2], and neuroplasticity [3,4]. Hence, studying these neural signals during sleep provides a critical window for understanding and improving brain health through the lens of sleep science.

Dopamine (DA) plays a pivotal role in sleep regulation, contributing uniquely to the modulation of sleep cycles and overall sleep architecture [5–7]. In the nucleus accumbens (NAc) of the brain, DA and D1 receptors promote wakefulness and alertness [8–10]. Enhanced dopaminergic activity in the NAc enhances wakefulness and reduces sleep needs, indicating a wake-promoting effect of DA in this region [9,11,12]. This arousal-promoting effect of DA may be related to its projections from the ventral tegmental area (VTA) [13,14]. In addition, the interplay between DA and other neurotransmitters regulates rapid eye movement (REM) and non-REM (NREM) sleep and influences sleep quality and duration [13,15,16]. The role of DA in the NAc emphasizes its importance in sleep regulation and offers key insights into the neurochemical mechanisms governing sleep and wakefulness.

However, existing techniques struggle to capture real-time neurotransmitter dynamics and single-neuron activity simultaneously. Traditional DA detection methods, such as microdialysis [17–19], require extensive sample preparation and suffer from slow response times [20,21]. Conversely, electrochemical techniques using microelectrode arrays (MEAs) provide immediate insight into neurotransmitter dynamics, enabling a deeper understanding of neural communication [22,23]. Integrating electrochemical methods into MEAs offers substantial advantages for the real-time monitoring of neurotransmitters, particularly for DA release [24,25], capturing dynamic changes within specific brain regions under various physiological and pathological conditions [26,27]. Moreover, MEAs can be enhanced with nanomaterials, such as platinum nanoparticles (PtNPs) [28], poly(3,4-ethylene dioxythiophene):poly(styrene sulfonate) (PEDOT:PSS) [29] combined with Nafion [30,31], or meta-phenylenediamine (mPD), which improve the affinity of the electrode for DA without requiring biological catalysts [32–34]. However, conventional modification methods rely on coating techniques that can inadvertently alter the electrical properties of other MEA sites [35], thus highlighting an urgent need for site-specific modification strategies to enhance the MEA performance in electrophysiological signal recording and neurotransmitter detection.

Despite substantial progress in electrode material development, current multimodal MEA systems remain fundamentally constrained by partial integration. As highlighted in recent studies [36–38], even advanced designs continue to rely on external Ag/AgCl reference electrodes implanted at anatomically distant sites, such as the posterior fontanelle. This externalized configuration introduces multiple limitations: It increases surgical complexity due to multi-site implantation, exacerbates tissue damage owing to the macroscopic size of the reference electrode [39,40], and undermines chronic measurement stability due to Ag/AgCl’s susceptibility to in vivo dissolution and potential drift [41,42]. Recent studies have explored iridium oxide (IrOx) as an alternative reference material to overcome these limitations, owing to its favorable electrochemical properties, including long-term potential stability, pH insensitivity, and compatibility with microfabricated on-probe integration [43–45]. These characteristics position IrOx as a promising candidate for enabling spatially integrated, chronically stable multimodal recordings in awake and naturally sleeping animals. This study addresses the aforementioned challenges by introducing advanced MultiTransm MEAs (MT MEAs) capable of simultaneously recording neuronal electrophysiological activity and detecting DA release. The MT MEA features a multichannel architecture that incorporates an integrated electrochemical 3-electrode system, thereby eliminating the need for external reference electrodes and enabling a compact, in vivo-compatible design. All electrode sites were functionalized with targeted nanocomposite coatings, enabling the high-fidelity acquisition of both electrophysiological and electrochemical signals, and achieving performance that surpasses that of conventional partially integrated MEAs. In vitro tests showed that the electrophysiological detection sites of the MT MEA exhibited excellent electrical properties, including low impedance and minimal phase delay. The reference electrode demonstrated long-term stability, while the DA detection sites displayed a low detection limit and high sensitivity. Coupled with in vivo sleep experiments, the MT MEA effectively captured physiological changes during sleep from multiple perspectives. This target-modified MT MEA thus emerges as a powerful instrument for directly investigating electrophysiological activity and neurotransmitter dynamics in living cells, providing valuable insights into the intricate dynamics of neurotransmission during sleep.

## Results

### Design and fabrication of MT MEAs

**Fig. 1.**
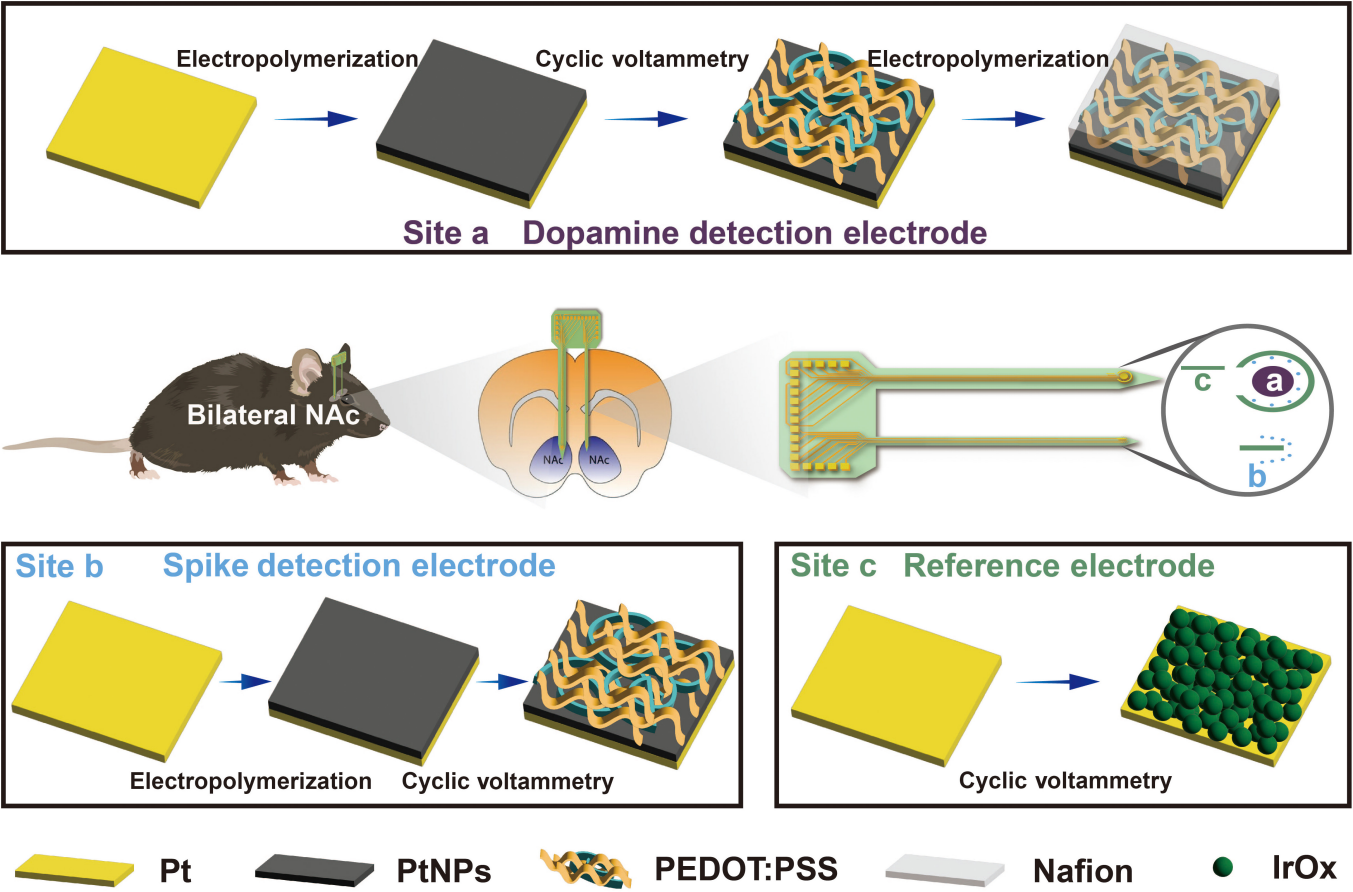
Schematic diagram of targeted modification in MultiTransm microelectrode arrays (MT MEAs). The working electrode for dopamine (DA) release detection is functionalized with a PtNP/PEDOT:PSS/Nafion composite. Electrodes for electrophysiological recordings are modified with PtNPs/PEDOT:PSS. The reference electrode, designed to maintain accurate potential measurement of the working electrode, is coated with IrOx.

A dual-needle architecture was designed for implantation into the left and right NAc to capture neural activity over a broader area. The architecture of the MT MEA is illustrated in Fig. 1. The array comprises 2 needles, each 5.70 mm long, with the left and right shanks measuring 350 μm and 200 μm in width, respectively. The 1.65-mm spacing between the shanks corresponds to the anatomical separation of the left and right NAc. Electrochemical detection is performed on one shank to minimize electrochemical interference and ensure stable signal recording, while the other supports electrophysiological and electrochemical recordings. The DA detection site was designed to spatially approximate the size of the NAc core, ensuring that the measured DA concentration is not biased by local microdomain fluctuations. In particular, the DA-sensing electrode, positioned on the left shank (site “a” in Fig. 1), features a diameter of 180 μm. An outer concentric ring surrounding the sensing site functions as the reference electrode, providing local shielding against environmental noise and improving signal stability. A rectangular platinum site (site “c” in Fig. 1) functions as the counter electrode in the 3-electrode system. The remaining sites (site “b” in Fig. 1) on both shanks are designated for electrophysiological recordings during wakefulness and sleep. Each recording site has a diameter of 20 μm and is spaced 90 μm apart. On the left shank, the electrophysiological recording sites adopt a radially symmetric array configuration to ensure precise spatial alignment with the central DA-sensing site. This facilitates accurate correlation between neurochemical and electrical signals. The MT MEAs were fabricated using micro-electromechanical systems (MEMS) technology (fig. S1).

### MT MEA surface targeted modification and electrical characterization

**Fig. 2.**
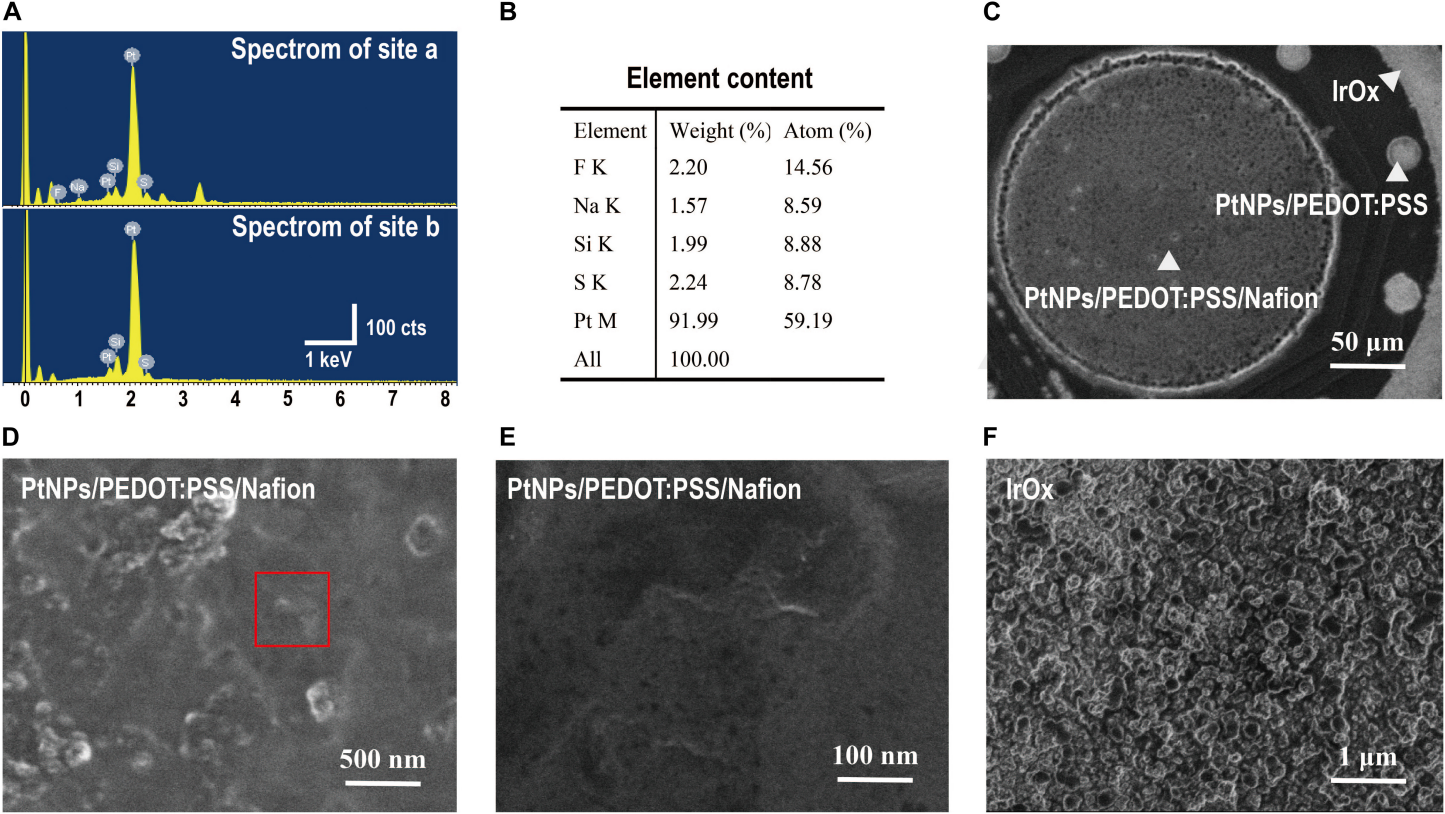
Results of targeted modifications on the integrated MT MEA. (A) Energy-dispersive spectroscopy of different sites. Site “a” corresponds to the working electrode for DA release detection, and site “b” corresponds to the electrophysiological detection electrodes. (B) Elemental analysis table of spectra from site “a”. (C to E) Scanning electron microscope (SEM) images of the working electrode modified with PtNPs/PEDOT:PSS/Nafion at magnifications of (C) 50 μm, (D) 500 nm, and (E) 100 nm. (F) SEM image of the reference electrode modified with IrOx at 1 μm.

Site-specific modification strategies were implemented to optimize the performance of electrophysiological and electrochemical detection sites. For electrophysiological detection sites (site “b” in Fig. 1), PtNPs/PEDOT:PSS was used to modify the electrodes, significantly reducing both impedance and phase. As shown in fig. S2, the impedance of bare electrode sites at 1 kHz was 1,268.53 ± 253.87 kΩ, which decreased to 3.93 ± 0.25 kΩ after PtNP modification and further to 1.48 ± 0.61 kΩ following PtNP/PEDOT:PSS modification. Similarly, the phase angle of the unmodified electrode sites was 72.42 ± 7.99°, decreasing to 19.74 ± 1.66° after PtNP modification and further to 11.21 ± 2.99° with PtNPs/PEDOT:PSS. However, PtNP/PEDOT:PSS/Nafion was applied at the DA release detection site to enhance its anti-interference capability. Selective electrochemical site modification was achieved through targeted electropolymerization to prevent the performance degradation of the electrophysiological detection sites and ensure the stability of the reference electrode. Energy-dispersive spectroscopy performed on all the electrode sites to detect fluorine, a characteristic element of Nafion, confirmed its selective incorporation at the electrochemical detection site (Fig. 2A). The fluorine content at the DA detection site was 14.56%, further verifying successful Nafion modification, as shown in the spectrum in Fig. 2B. Fluorine was detected only at the Nafion-modified electrode site, with no presence at other sites. Scanning electron microscope (SEM) images of the modified sites are presented in Fig. 2C to E. An additional representative impedance map is provided in fig. S3, showing that the unmodified recording sites maintain their expected low-impedance characteristics. This result further supports the spatial selectivity of the surface modification strategy and confirms the electrical isolation of the Nafion-functionalized electrochemical channels from adjacent electrophysiological sites. Furthermore, to enhance the stability of the reference electrode, IrOx was applied as a modification [46–48], with the corresponding SEM image shown in Fig. 2F.

### In vitro DA detection performance of MT MEAs

**Fig. 3.**
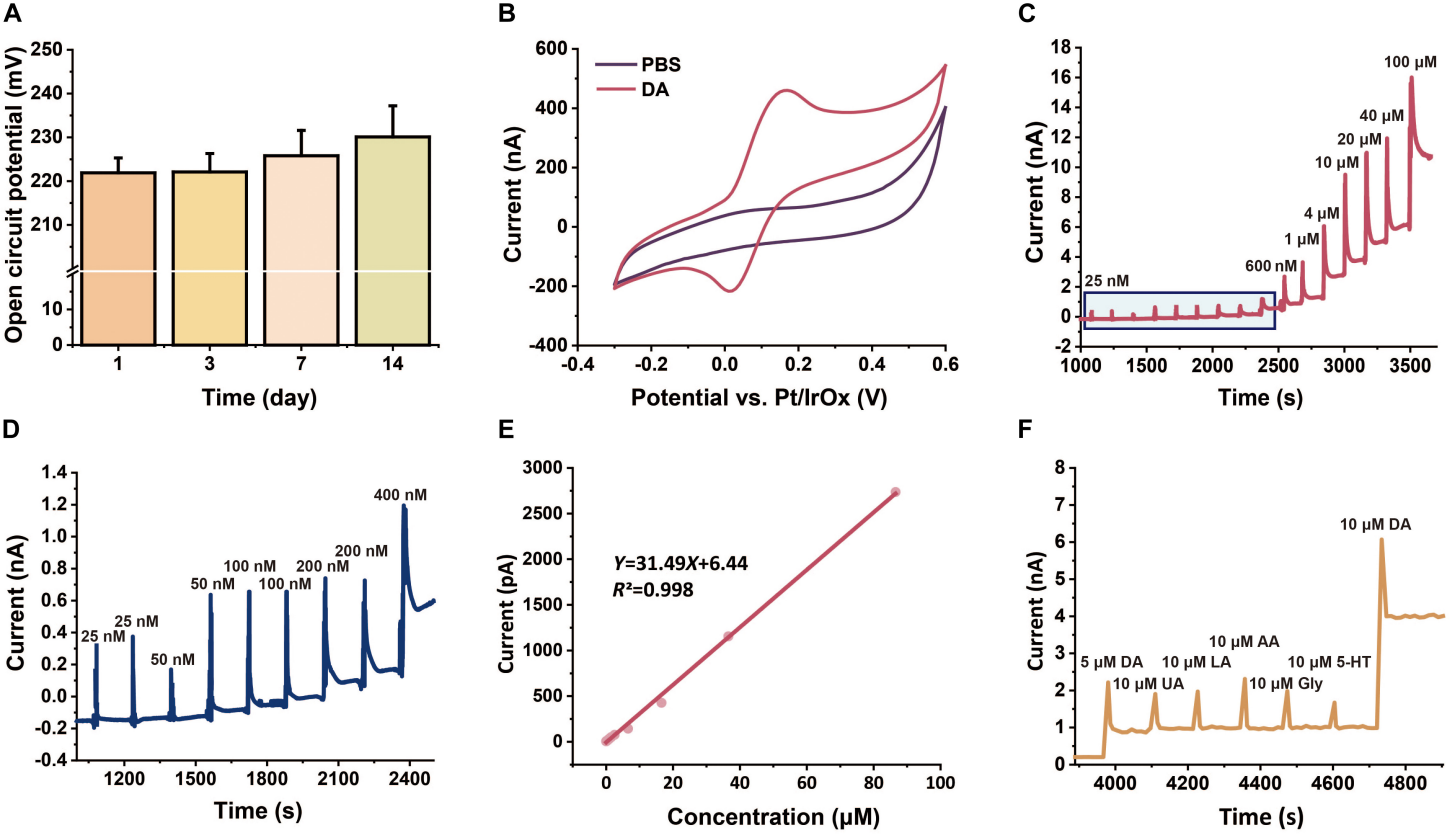
Electrical performance of the MT MEA and in vitro calibration for DA release detection. (A) Open-circuit potential of the IrOx-modified reference electrode over a 14-d period. (B) Cyclic voltammetry (CV) scans in PBS and DA-containing solution to identify the DA oxidation peak (−0.3 to 0.6 V, 100 mV/s). (C and D) MT MEA responses in varying concentrations of DA solutions. (E) Linear fit of the MT MEA response as a function of DA concentration. (F) MT MEA responses to DA and potential interfering substances.

In vitro tests were conducted to validate the effectiveness of the electrode in detecting DA. As shown in Fig. 3A, the IrOx-modified reference electrode maintained a stable open-circuit potential (~220 mV) over 14 d post-implantation (days 1, 3, 7, and 14), with fluctuations within ±20 mV, confirming its long-term electrochemical stability and suitability for in vivo monitoring. In a phosphate-buffered saline (PBS) solution, cyclic voltammetry (CV) scans showed no oxidation peaks. However, when immersed in a 200 mM DA solution, an oxidation peak appeared at 0.17 V, as shown in Fig. 3B, suggesting that this lower oxidation potential may be advantageous for both electrode material integrity and the biological environment. Next, chronoamperometry (CA) was employed to calibrate DA concentrations between 25 nM and 100 μM, with the results presented in Fig. 3C and D. Calibration data demonstrated a detection limit as low as 25 nM, with a sensitivity of 31.49 pA/μM (Fig. 3E). An anti-interference test was conducted to confirm that DA release was not compromised by the interfering substances in vivo (Fig. 3F). By adding 5 μM DA, 10 μM uric acid (UA), 10 μM lactic acid (LA), 10 μM ascorbic acid (AA), 10 μM serotonin [5-hydroxytryptamine (5-HT)], 10 μM glycine (Gly), and 10 μM DA into the solution, the steady-state current change indicated that the electrode exhibited the highest response to DA, significantly surpassing its responses to the other substances. These electrochemical tests confirmed the high stability, low impedance, high sensitivity, and strong anti-interference capability of the electrode, making it suitable for long-term, precise electrochemical measurements.

### In vivo detection of electrophysiological signals and DA release during the sleep–wake cycle

**Fig. 4.**
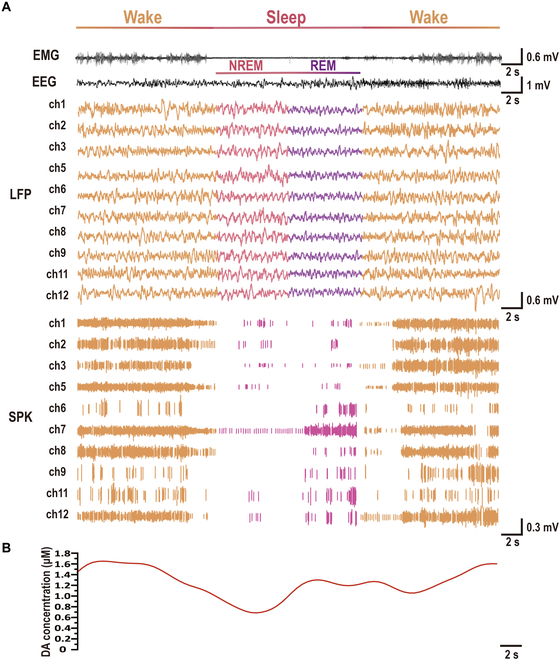
In vivo neural activity and corresponding DA fluctuations across wake, non-rapid eye movement (NREM), and REM measured with the MT MEA. (A) Simultaneous in vivo electrophysiological recordings at the cellular level, including spikes and local field potentials (LFPs), alongside electroencephalography (EEG) and electromyography (EMG) signals, across wake, NREM, and REM sleep. (B) Corresponding DA fluctuations in the nucleus accumbens (NAc) during wake, NREM, and REM sleep.

After validating the electrophysiological and electrochemical detection performances in vitro, in vivo experiments were conducted to measure the electrophysiological signals and DA release simultaneously. Before electrode implantation into the NAc, in vivo calibration was performed following methodologies consistent with those previously described [49]*.* As shown in the revised fig. S4, no discernible DA oxidation peak was observed near 0.17 V. The absence of a detectable DA signal under these conditions provides strong evidence supporting the chemical specificity of our electrochemical detection strategy. Subsequently, an integrated 3-electrode system was implanted in the NAc along with electroencephalography (EEG) and electromyography (EMG) electrodes, enabling real-time monitoring of electrophysiological and electrochemical signals during sleep. As shown in Fig. 4A, sleep is divided into different stages based on the EEG and EMG recordings [50]. Single-neuron spike activity and the corresponding local field potentials (LFPs) were analyzed across sleep stages. Spike firing patterns varied noticeably, with higher firing frequencies observed during wakefulness. The LFP in the NAc also exhibited distinct patterns: LFP signals were densely distributed during wakefulness, whereas during NREM sleep, the amplitudes were larger at lower frequencies. In REM sleep, amplitudes were smaller and frequencies were lower than those in wakefulness, consistent with the results reported in previous studies [51,52]. As illustrated in Fig. 4B, fluctuations in DA release in the NAc were recorded synchronously with neural activity, revealing a dynamic relationship between DA levels and sleep states.

### REM-associated neuronal dynamics and LFP–EEG spectral comparison across sleep stages

**Fig. 5.**
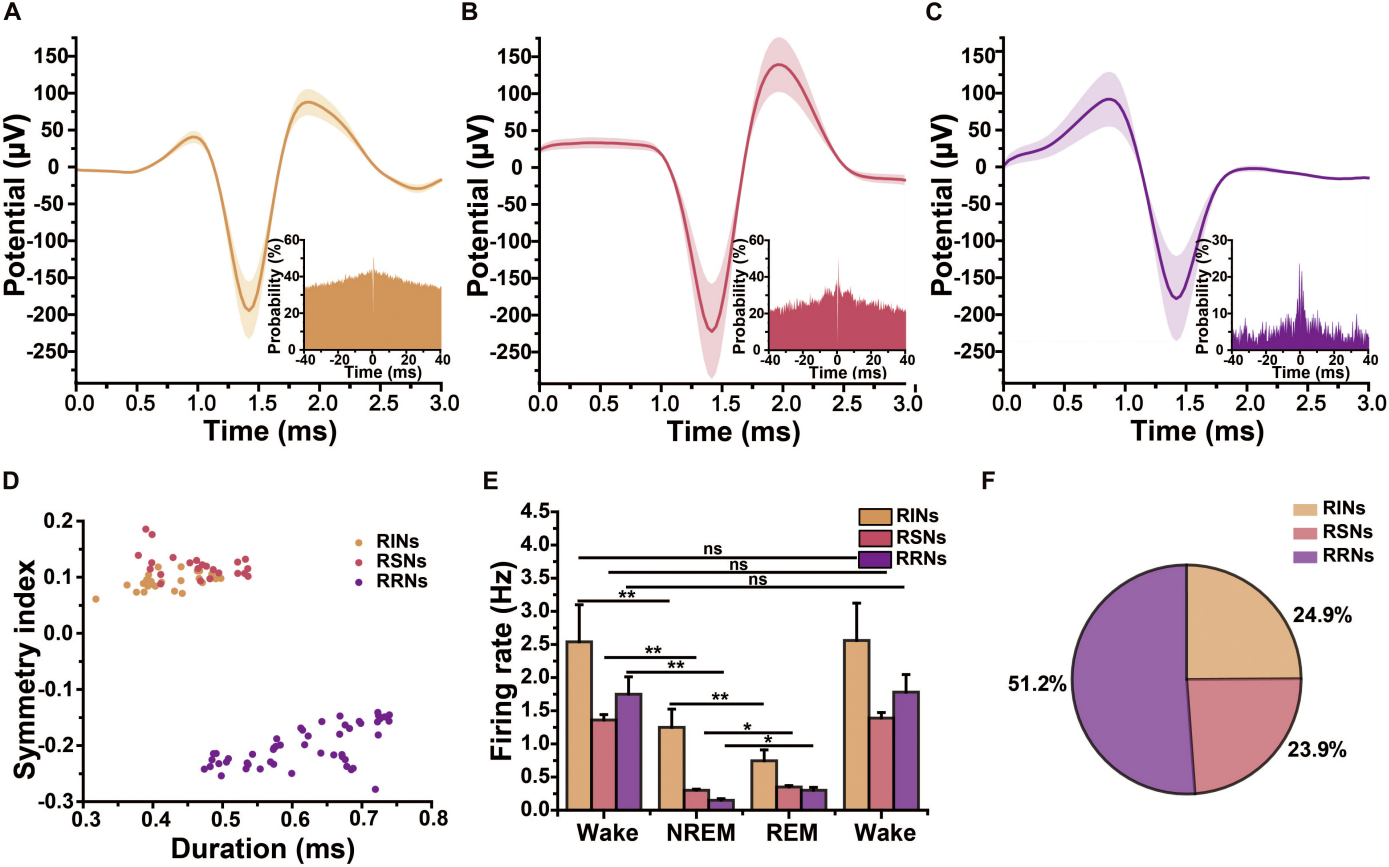
MT MEA reveals distinct cell-level activity across wake, NREM, and REM sleep. (A to C) Spike waveforms and their autocorrelation statistics. (D) Distinction among 3 neuron types using *k*-means clustering, including REM-inactive neurons (RINs), REM-stable neurons (RSNs), and REM-rhythmic neurons (RRNs). (E) Average firing rates of 3 neuron types across wake, NREM, REM, and wake. (F) Proportions of the 3 neuron types among all detected neurons across wake, NREM, and REM. *n* = 6 mice, *P < 0.05, **P < 0.01.

Figure 5 summarizes the detected spike waveforms and the corresponding firing rate changes across different types of neurons. Three distinct neuronal waveforms were identified, as shown in Fig. 5A to C. An autocorrelogram analysis revealed that REM-rhythmic neurons (RRNs) displayed a sharp peak within 10 ms. By contrast, the firing peaks of the other 2 neurons were more dispersed, suggesting that RRNs have highly rhythmic and high-frequency firing patterns, particularly during REM sleep. As shown in Fig. 5D, the pulse widths and symmetry [53] of the 97 detected neurons were analyzed and clustered into REM-inactive neurons (RINs), REM-stable neurons (RSNs), and RRNs using the *k*-means algorithm. Examination of firing rate changes (Fig. 5E) revealed that RINs had the highest firing rate during wakefulness and the lowest during REM sleep. By contrast, RSNs and RRNs exhibited the highest firing rates during wakefulness and the lowest during NREM sleep. Neuron proportion analysis revealed that RRNs were the most abundant, accounting for 51.20% of the detected neurons, followed by RINs (24.90%) and RSNs (23.90%) (Fig. 5F). These results suggest that pyramidal neurons, predominant in the NAc, are the primary neurons associated with sleep regulation.

**Fig. 6.**
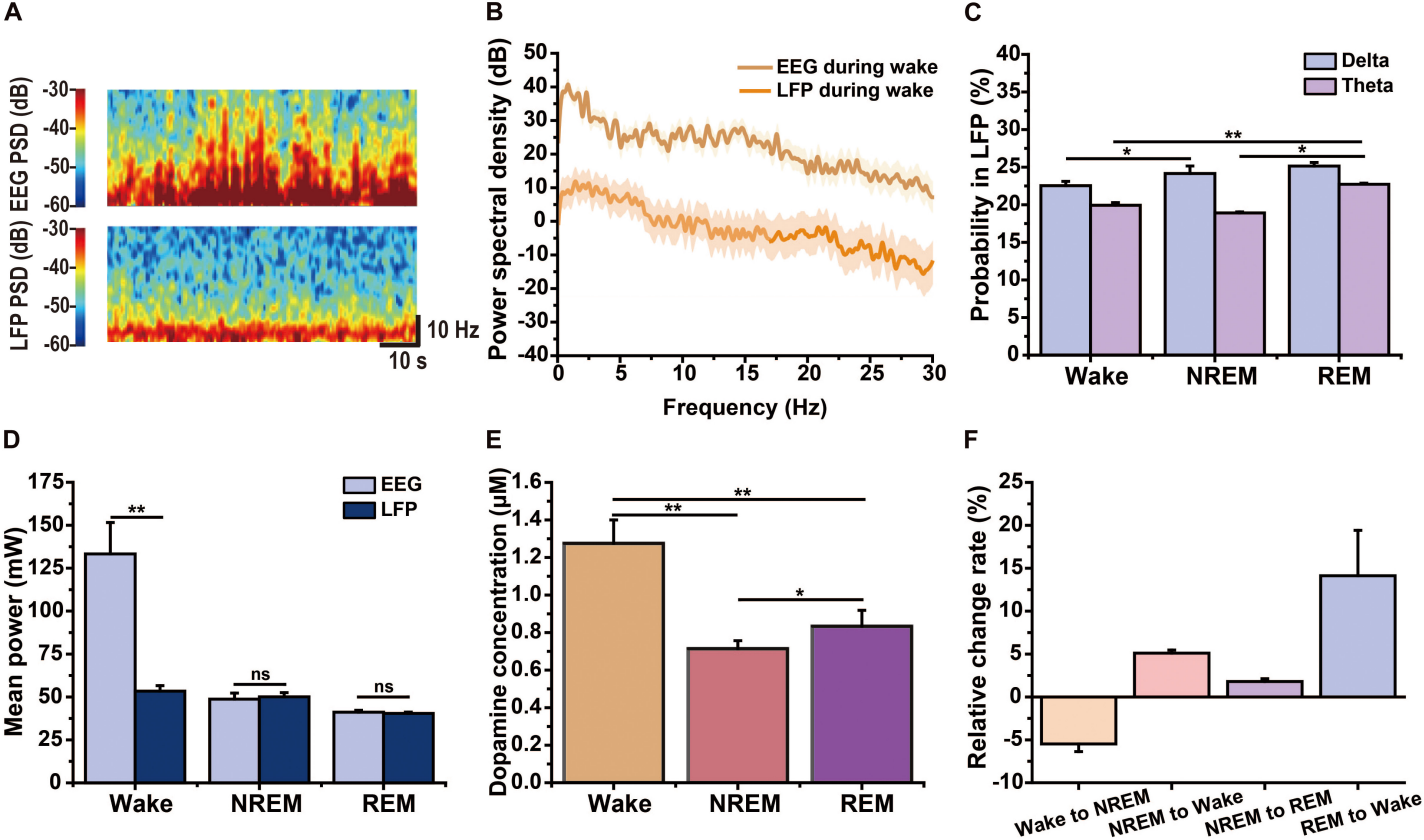
Electrical activity at different brain depths and DA concentrations in the NAc across wakefulness and sleep stages. (A) Spectral patterns of EEG and NAc LFP signals during wakefulness. (B) Power spectral density of EEG and NAc LFP during wakefulness. (C) Probability distribution of delta (0 to 4 Hz) and theta (4 to 8 Hz) power in LFPs across wakefulness and sleep stages. (D) Mean power of EEG and LFP across wake, NREM, and REM sleep. (E) Average DA concentration across wake, NREM, and REM. (F) Relative changes in DA concentration across wakefulness and sleep stages. *n* = 6 mice, *P < 0.05, **P < 0.01.

We analyzed the spectral density variations across sleep stages and frequency bands to investigate the differences between LFPs and EEG. The power spectra of the LFPs and EEG during wake are displayed in Fig. 6A. As shown in Fig. 6B, EEG power spectral density was significantly higher than NAc LFP power during wakefulness. However, no significant differences were observed between the NREM and REM sleep groups (fig. S4). Further analysis of specific frequency bands revealed notable delta (0 to 4 Hz) and theta (4 to 8 Hz) variations across the sleep stages. In the EEG, delta power was most dominant during NREM, while theta power was highest during REM (fig. S5), consistent with the results reported in previous studies [54]. In LFPs, delta power increased during the transition from wakefulness to NREM, whereas theta power remained the highest during REM (Fig. 6C). As shown in Fig. 6D, an analysis of the average power across sleep stages revealed that EEG power during wakefulness (2,665.59 ± 366.13 mW) was significantly higher than LFP power (1,067.93 ± 64.00 mW, **P < 0.01). However, no significant differences were observed during NREM or REM sleep, suggesting that the NAc plays a more prominent role in sleep-related processes.

The DA concentrations were also analyzed across different sleep stages, as shown in Fig. 6E. During wakefulness, DA concentration was highest at 1.27 ± 0.12 μM, while the lowest level was observed during NREM sleep at 0.71 ± 0.04 μΜ (*P < 0.05, **P < 0.01). The relative rate of change in DA concentration during sleep-stage transitions was also examined (Fig. 6F). Four distinct transition types were identified, consistent with previous studies [55,56]. From wakefulness to NREM, DA concentration decreased by 5.48 ± 0.89%, while it increased by 5.10 ± 0.35% from NREM to wakefulness. A modest 1.80 ± 0.32% increase was observed during the NREM to REM transition. The most pronounced rise in DA concentration occurred during the REM-to-wake transition, with an increase of 14.12 ± 5.30%. These findings align with the observed changes in spike activity, suggesting that RSNs and RRNs may be temporally modulated by, or synchronized with, dopaminergic signaling. This study further supports the role of DA in the NAc as a central regulator of the sleep–wake cycle, emphasizing its importance in maintaining wakefulness and modulating sleep dynamics.

## Discussion and Conclusion

In this study, we designed an MT MEA with an integrated 3-electrode system. The electrophysiological detection sites were modified with PtNPs/PEDOT:PSS to enhance conductivity and increase the specific surface area. The reference electrode was coated with IrOx to ensure long-term stability and improve the potential performance repeatability of the electrode during in vivo monitoring. The electrochemical detection sites were selectively modified with PtNPs/PEDOT:PSS/Nafion, enhancing biocompatibility and enabling DA release detection. By employing a directional surface modification strategy, we achieved selective enhancement of electrochemical sensitivity without interfering with adjacent electrophysiological sites. Compared with conventional uniform coating or localized drop-casting methods, this approach simplifies the fabrication process (Table S1) while preserving the spatial specificity and long-term functional stability necessary for dual-mode recording (Table S2).

After site-specific modifications, the integrated 3-electrode MT MEA was implanted in the NAc of mice to enable the simultaneous monitoring of electrophysiological activity and DA release. This study revealed the coordinated changes in single-neuron firing activity and DA concentrations across different sleep stages. Three distinct neuron types were identified. Among them, RSNs and RRNs exhibited the highest firing rates during wakefulness and the lowest during NREM sleep, mirroring DA release levels, which were highest during wake and lowest during NREM. These results suggest that RSNs and RRNs may be functionally modulated by, or temporally synchronized with, dopaminergic signaling. However, their precise neurochemical identity remains to be validated by future studies involving genetic labeling, pharmacological manipulation, or electrophysiological classification with ground truth references. In addition, a comparative analysis of EEG and NAc LFP signals revealed that LFP patterns in the NAc closely resembled EEG signals during NREM and REM sleep but differed significantly during wakefulness. This suggests that the NAc may be critical in sleep-stage transitions. Although formal power analysis was not performed in this study, future investigations would benefit from incorporating a priori power calculations to improve the robustness of the findings.

In summary, the MT MEA represents a fully integrated, miniaturized MEA that eliminates the need for external reference electrodes by incorporating an on-probe, IrOx-based 3-electrode system. Through targeted nanocomposite modifications applied to the working, reference, and recording sites, this platform enables the simultaneous detection of DA dynamics and neural electrical activity. In addition, its enhanced biocompatibility and electrochemical stability support long-term in vivo implantation, making it a valuable tool for dissecting neurochemical and electrophysiological processes during sleep and advancing multimodal investigations in deep brain circuits. While the current system achieves sub-second temporal resolution, future studies targeting faster neuromodulatory events—such as spindle-locked DA transients—may benefit from incorporating optimized fast-scan cyclic voltammetry (FSCV) approaches with higher temporal precision. Overall, the study findings offer new insights into the physiological regulation of sleep.

## Materials and Methods

### Reagents and apparatus

DA was sourced from Acros Organics (Belgium), Nafion was provided by MACKLIN (China), and EDOT was provided by Aladdin (China). All other chemicals were purchased from Sigma (USA). The chemical species were monitored using a potentiostat (Princeton Applied Research VersaSTAT4, USA), and electrical signals were recorded using a 128-channel neural data acquisition system (Blackrock Microsystems, USA).

### Fabrication of the MT MEAs

The MT MEA was fabricated on a silicon-on-insulator (SOI) silicon wafer using MEMS technology. First, an oxide insulation layer was deposited by low-pressure chemical vapor deposition. A conductive layer (Ti/Pt) was then deposited following photolithography and development with an AZ5214 negative photoresist, followed by a lift-off. Subsequently, plasma-enhanced chemical vapor deposition was used to deposit 300 nm of silicon oxide and a 500-nm silicon nitride layer (Si₃N₄) as insulation layers. Next, photolithography with an AZ1500 photoresist was employed to etch windows in the silicon nitride to expose the electrode sites. The silicon and top thermally oxidized layer were selectively etched to define the MEA shape. Finally, the electrodes were detached from the SOI by wet etching.

### Targeted modification of the MT MEAs

Targeted modifications were applied to the electrophysiological detection sites, neurotransmitter detection sites, and reference electrodes to enhance electrode detection performance. First, electrochemical deposition was performed on all electrode sites, except the reference electrode, by immersing the electrodes in a platinum black plating solution and using CA (−0.95 V, 60 s). For electrochemical detection, site modification with PEDOT:PSS and CV (0 to 0.95 V, 100 mV/s, 6 cycles) was conducted in a 4-ml plating solution containing 0.1 M PSS and 20 mM EDOT. The electrode tip was immersed in a 5% (w/v) Nafion solution for deposition to further enhance the DA selectivity. Multiple rounds of CA deposition (1.0 V, 60 s per syringe) were performed, with a 5-min interval between cycles, during which the electrodes were rinsed with deionized water to prevent cross-contamination. This stepwise deposition method ensured a uniform Nafion coating and optimized electrochemical site performance. Finally, to improve the in vivo stability, the reference electrode was modified with IrOx by CV electrodeposition in an iridium plating solution (0.05 to 0.55 V, 100 mV/s, and 100 cycles) [48]. After incubation at room temperature for 72 h, a highly sensitive and long-term stable MT MEA was successfully fabricated.

### In vitro testing methods

Prior to in vivo implantation, the MT MEA was subjected to in vitro testing and calibration under conditions mimicking the brain microenvironment. In vitro DA calibration experiments were performed in PBS (pH 7.4) at 37 °C. The MEA was placed in a 10 μM DA solution to identify the appropriate oxidation peak for calibration potential. The IrOx-modified sites on the array functioned as the reference electrode, whereas the platinum electrode was the counter electrode; calibration was performed using 10 ml of pure saline solution. Low analyte concentrations were added incrementally to determine the limit of detection until the signal-to-noise ratio (SNR) reached at least 3. Sensitivity was assessed thrice by introducing 10 μM of the analyte into the solution. Finally, selectivity was evaluated by ensuring that the recording sites did not interfere with each other. This was verified by adding 10 μM of AA, UA, 3,4-dihydroxyphenylacetic acid (Dopac), serotonin (5-HT), and Gly.

### Animal surgery and in vivo detection

C57BL/6J mice (30 g, *n* = 6, 6 weeks) were obtained from Vital River (Beijing, China). The Institutional Animal Care and Use Committee of the Aerospace Information Research Institute of the Chinese Academy of Sciences approved all the animal experiments. The MT MEA was surgically implanted bilaterally into the NAc of each mouse. Briefly, the mice were anesthetized using the RWD520 isoflurane anesthesia system and secured in a stereotaxic frame. Craniotomies were performed to target the NAc [anterior-posterior (AP): 1.10 mm, medial-lateral (ML): ±1.00 mm, dorsal-ventral (DV): −4.75 mm]. The mice were allowed to recover for at least 7 d after surgery. Subsequently, the recording system was connected to the MEA to simultaneously record multiple signals over 24 h, capturing both sleep and activity states.

### Processing and statistics of neural information

Neural signals were recorded as the animals moved freely. The MT MEA was interfaced with a Princeton electrochemical workstation for electrochemical recordings sampled at 50 Hz and simultaneously connected to an electrophysiological recording system (Blackrock Microsystems, USA) for neural signal acquisition at a sampling rate of 30 kHz. Raw signals were separated into spike and LFP components using high-pass (>200 Hz) and low-pass (<200 Hz) filters. Spike and LFP signals were analyzed using NeuroExplorer (Nex Technologies, CO, USA). Spike sorting was performed by detecting events that crossed a fixed negative threshold of 3 standard deviations. *K*-means clustering combined with an adaptive expectation–maximization algorithm was applied to classify neuronal activity, and cluster validity was assessed using the silhouette score, gap statistic, and Davies–Bouldin index. All 3 metrics consistently identified *k* = 3 as the optimal number of clusters, supporting the classification of 3 distinct spike types (fig. S10). Only units with an SNR greater than 4 were retained for further analysis. Sleep stages were classified based on EEG and EMG recordings, using standard criteria: Wakefulness was marked by low-amplitude, high-frequency EEG and high EMG tone; NREM by high-amplitude, low-frequency EEG and reduced EMG; and REM by low-amplitude, mixed-frequency EEG with EMG atonia [50]. All data are presented as mean ± standard error. Statistical significance was assessed using analysis of variance (ANOVA) with Tukey’s post hoc test. A *P* value less than 0.05 was considered significant. The formula for calculating the relative change in DA release from stages *A* to *B* is as follows:

$$\Delta_{{\mathrm{DA}}_{A->B}}=\frac{{\mathrm{DA}}_{B}-{\mathrm{DA}}_{A}}{{\mathrm{DA}}_{A}}\times 100\%$$ (1)

This equation quantifies the percentage change in DA levels between the 2 stages.

## Data Availability

All data included in this study are available upon request by contacting the corresponding author.
