# Supplementary material for: Targeted-Modified MultiTransm Microelectrode Arrays Simultaneously Track Dopamine and Cellular Electrophysiology in Nucleus Accumbens during Sleep–Wake Transitions
Source: Research (Wash D C). 2025 Oct 9;8:0944. doi: 10.34133/research.0944 (PMC12509213; doi:10.34133/research.0944)
Supplement: Supplementary 1 — Figs. S1 to S10 Tables S1 and S2 [file research.0944.f1.zip › Supplementary Information.docx]

Title

Targeted-Modified MultiTransm MEAs Simultaneously Track Dopamine and Cellular Electrophysiology in NAc During Sleep–Wake Transitions

**Authors**

Qianli Jia^1,2^, Zhaojie Xu^1,2^, Yu Wang^1,2^, Yiming Duan^1,2^, Yu Liu^1,2^, Jin Shan^1,2^, Jiale Ma^3^, Qi Li^4^, Jinping Luo^1,2^, Yan Luo^4^, Ying Wang^4^, Shumin Duan^3^, Yanqin Yu^3*^, Mixia Wang^1,2*^ and Xinxia Cai^1,2*^†

**Affiliations**

Qianli Jia, Zhaojie Xu, Yu Wang, Yiming Duan, Yu Liu, Jin Shan, Jiale Ma, Jinping Luo, Mixia Wang^*^ and Xinxia Cai^*^

1.State Key Laboratory of Transducer Technology, Aerospace Information Research Institute, Chinese Academy of Sciences, Beijing 100190, China.

2.University of Chinese Academy of Sciences, Beijing 100049, China.

^*^Address correspondence to: [xxcai@mail.ie.ac.cn](mailto:xxcai@mail.ie.ac.cn), [wangmixia@mail.ie.ac.cn](mailto:wangmixia@mail.ie.ac.cn)

Jiale Ma, Shumin Duan and Yanqin Yu*

3.NHC and CAMS Key Laboratory of Medical Neurobiology, MOE Frontier Science Center for Brain Research and Brain-Machine Integration, School of Brain Science and Brain Medicine, Zhejiang University, Hangzhou, China

^*^Address correspondence to: [yanqinyu@zju.edu.cn](mailto:yanqinyu@zju.edu.cn)

Qi Li, Yan Luo and Ying Wang

4.Department of Anesthesiology, Ruijin Hospital, Shanghai Jiaotong University School of Medicine, Shanghai 200025, China


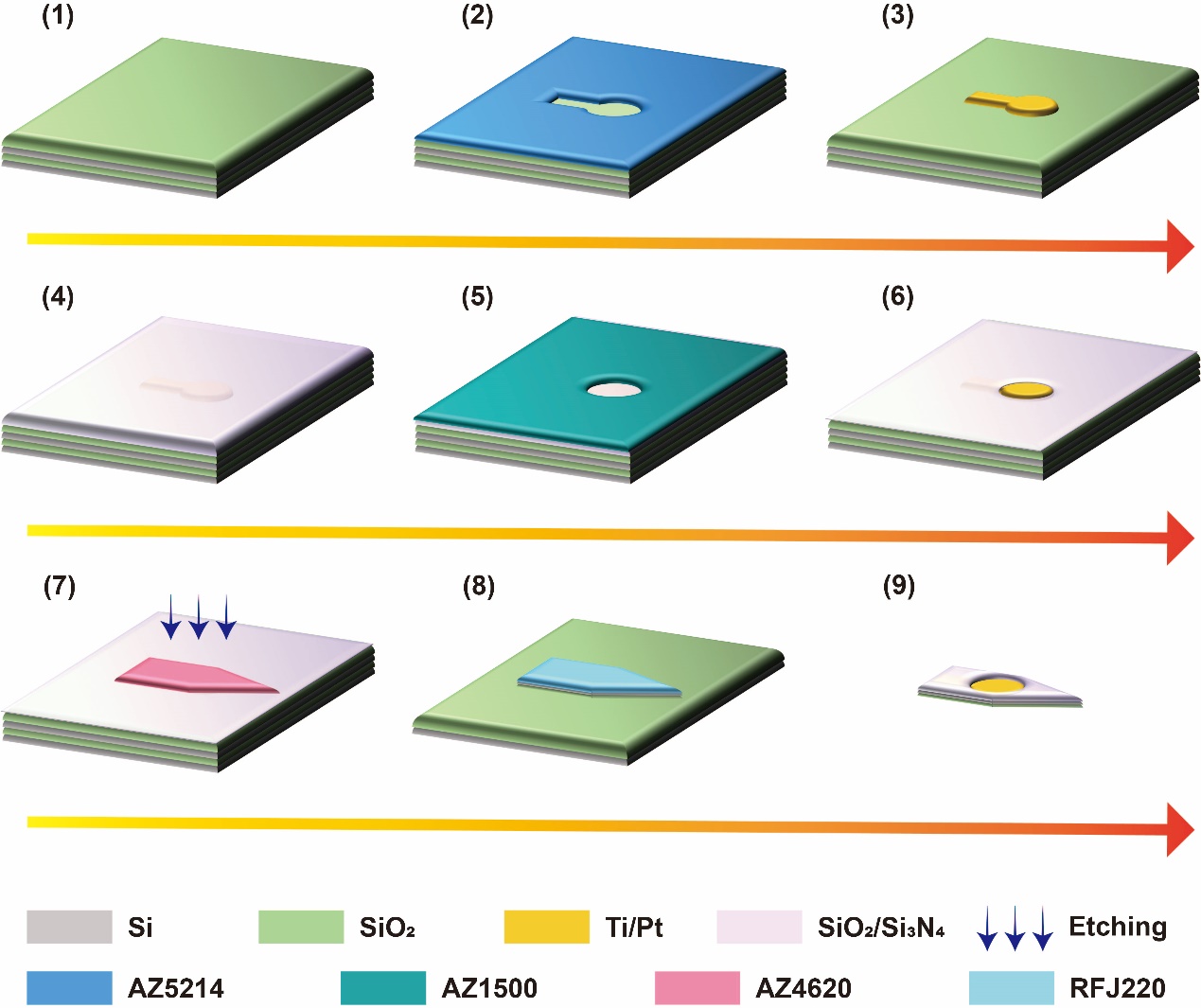


**Supplementary Figure S1.** Schematic diagram of the fabrication process of MultiTransm microelectrode arrays (MT MEAs): (1) Thermal oxidation on SOI to produce a layer of SiO_2_. (2) Generate the photoresist patterning for metal trace by photolithography (5214E) and sputtering metal layer (Ti/Pt). (3) Pattern metal layer by lift-off. (4) Deposition of SiO_2_/Si_3_N_4_ by plasma-enhanced chemical vapor deposition. (5) Generate the photoresist patterning for the exposed site by photolithography (AZ1500). (6) Insulation layer (SiO_2_/Si_3_N_4_) etching. (7) Generate the photoresist patterning for the shape of the MT MEAs by photolithography (AZ4620). (8) SiO_2_/Si layer etching. (9) Releasing of the MT MEAs.


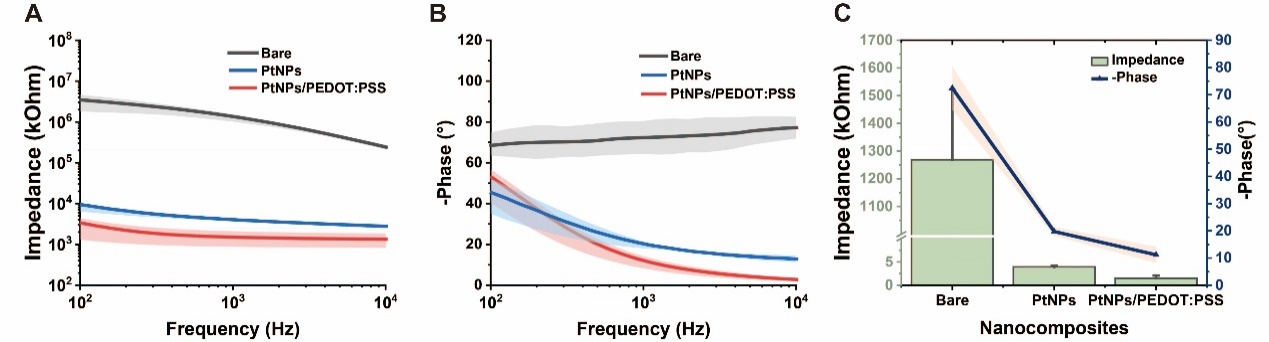


**Supplementary Figure S2.** Electrical performance characterization of PtNPs/PEDOT:PSS-modified MultiTransm microelectrode arrays (MT MEAs). **A** Impedance characteristics of bare, PtNPs and PtNPs/PEDOT:PSS-modified MT MEAs at different frequencies. **B** Phase characteristics of bare, PtNPs, and PtNPs/PEDOT:PSS-modified MEAs at different frequencies. **C** The average impedance and phase of bare, PtNPs, and PtNPs/PEDOT:PSS-modified-MEAs at 1 kHz frequency (n = 6).


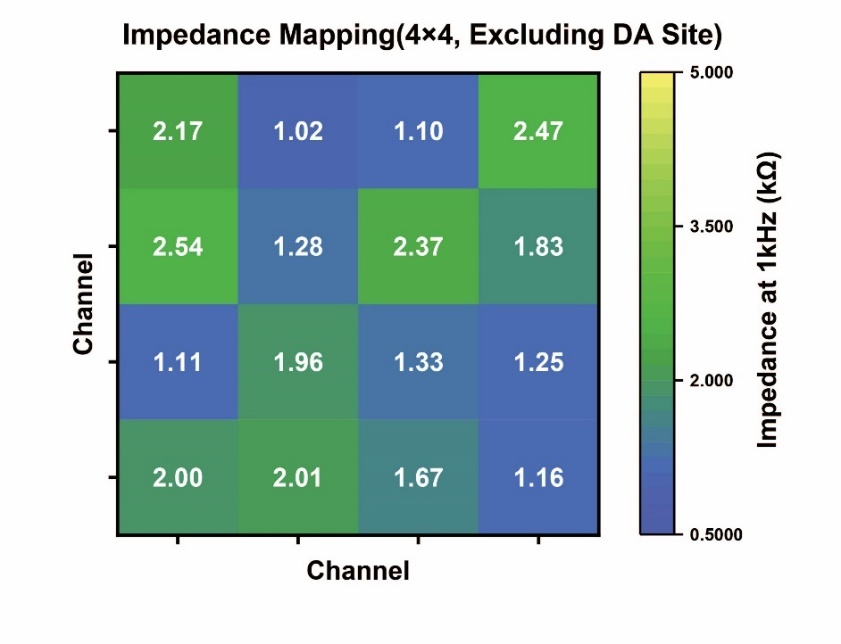


**Supplementary Figure S3.** Impedance mapping of the 16-channel array at 1 kHz following Nafion-based site-specific surface modification, confirming that the modification process preserves electrical isolation between electrochemical and electrophysiological channels without inducing crosstalk.


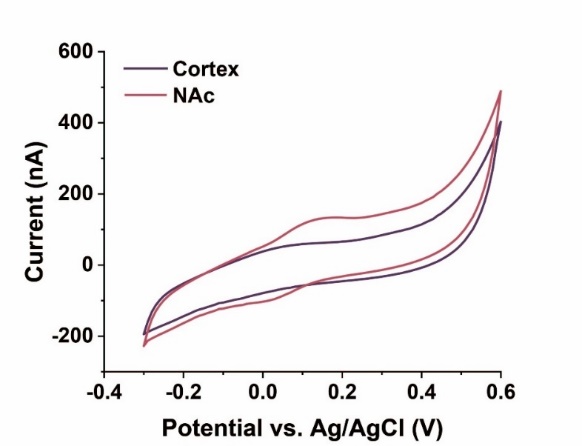


**Supplementary Figure S4.** Representative cyclic voltammetry (CV) waveforms recorded from cortical and nucleus accumbens (NAc) sites. Cortical sites exhibit no detectable dopamine redox peaks around 0.17 V, in contrast to the characteristic oxidation signal observed in the NAc.


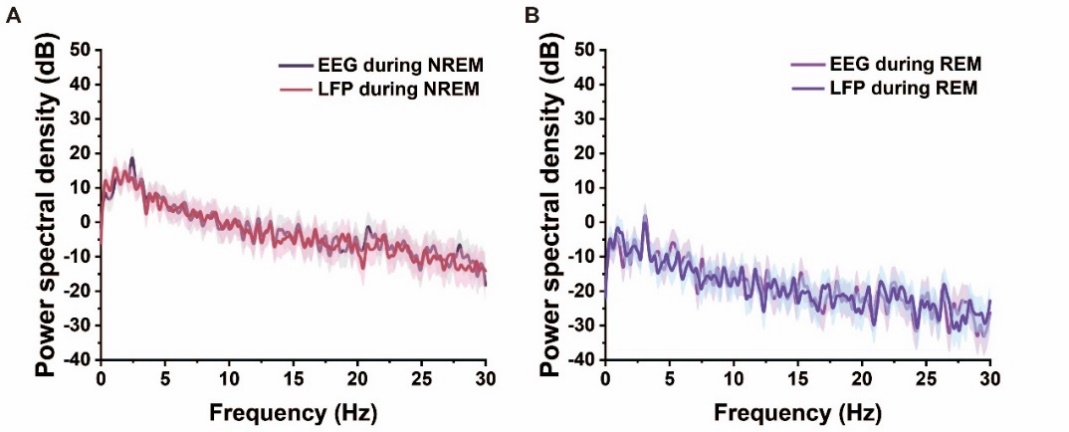


**Supplementary Figure S5.** Electrical activity at different depths of the brain during non-rapid eye movement (NREM) and REM. **A** Power spectral density of electroencephalography (EEG) and local field potential (LFP) in the nucleus accumbens (NAc) during NREM. **B** Power spectral density of EEG and LFP in the NAc during REM.


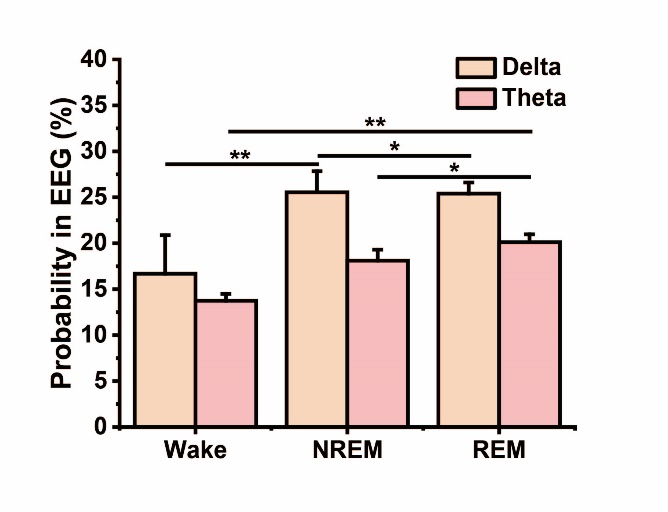


**Supplementary Figure S6.** Probability of delta and theta in electroencephalography (EEG) power in the delta and theta bands.


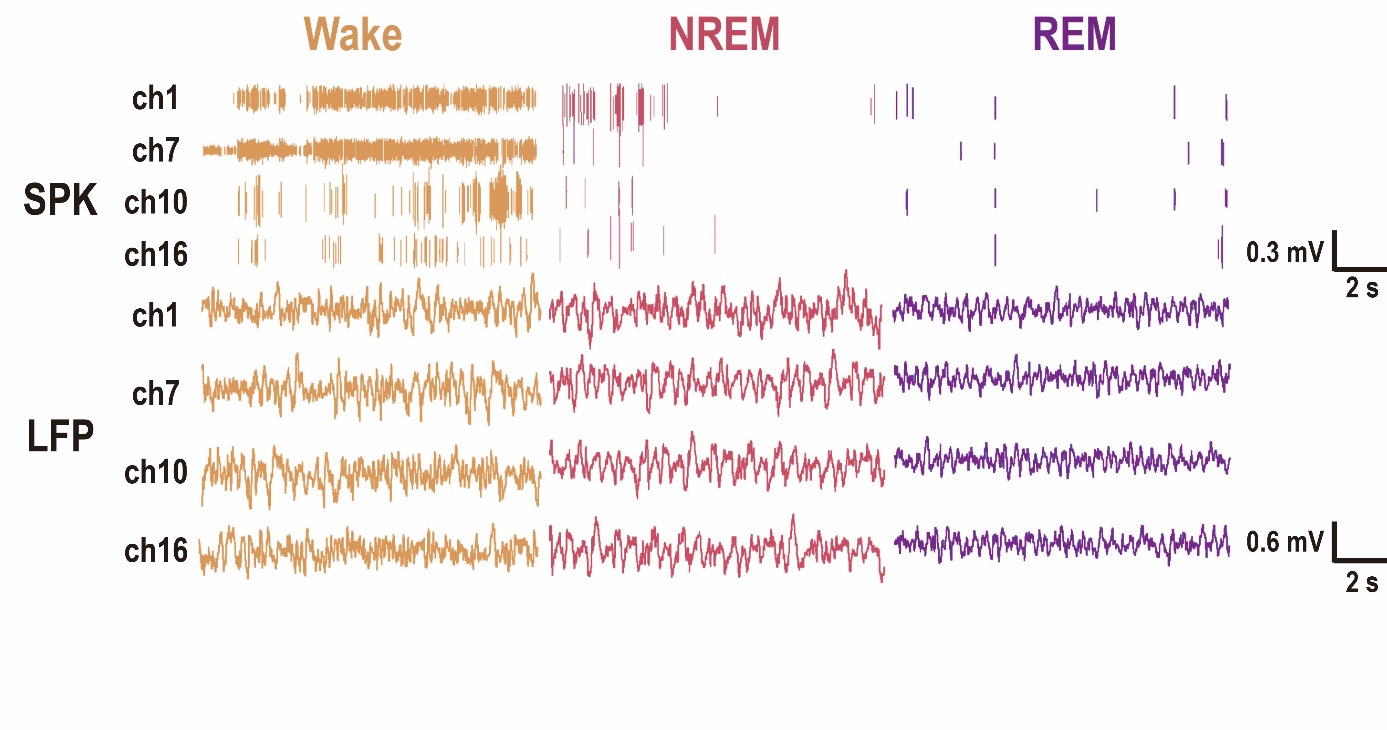


**Supplementary Figure S7.** **Electrophysiological measurements across different brain regions: ipsilateral recordings (channels 1 and 16) and contralateral recordings (channels 7 and 10).**


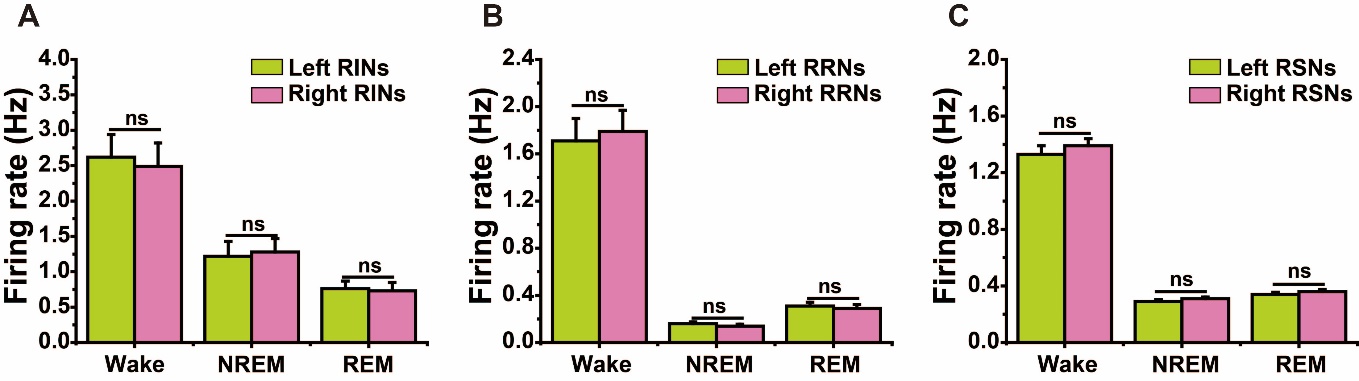


**Supplementary Figure S8.** **Comparison of firing rates among different spike neuron types in bilateral brain regions: A. RINs, B. RRNs, and C. RSNs. REM: rapid eye movement; RINs:** REM-inactive neurons; RRNs: REM-rhythmic neurons; RSNs: REM-stable neurons


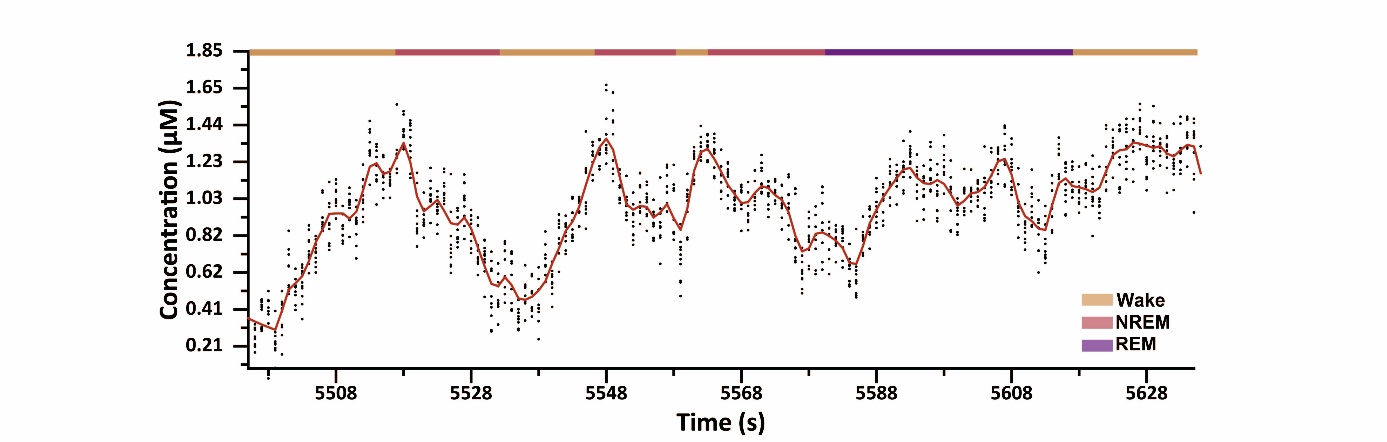


**Supplementary Figure S9.** **Dopamine monitoring during prolonged sleep–wake cycles.**

Table S1 Comparison of Electrode Surface Modification Strategies for Selective Electrochemical Sensing and Multimodal Compatibility

| **Nanocomposites** | **Deposition** | **Measure** | **Number of implanted electrodes for electrochemistry** | **Reference** |
| --- | --- | --- | --- | --- |
| GCF-MEAs | - | FSCV | 3 | ^[1]^ |
| PEDOT/CNT | CC | SWV | 3 | ^[2]^ |
| Enzyme/CNT | Drop casting and I-T | CPA | 3 | ^[3]^ |
| Pt/rGO/Nafion-MEAs | Drop casting | CPA | 3 | ^[4]^ |
| Pt/Ir/Nafion | Dip coating | CPA | 2 | ^[5]^ |
| CFM/Pt/Nafion | Dip coating | CPA | 2 | ^[6]^ |
| This work | CV and I-T | CPA | 1 |  |

* GCF—Glassy carbon fiber-like; MEAs—Microelectrode arrays; FSCV—Fast-scan cyclic voltammetry; PEDOT—Poly (3,4-ethylene dioxythiophene); CC—Chronocoulometry; SWV—Square wave voltammetry; CNT—Carbon nanotube; I-T—Chronoamperometry; CPA— Constant-potential amperometry; Pt—Platinum; rGO—Reduced graphene oxide; Ir—Iridium; CFM—Carbon fiber microelectrode; CV—Cyclic voltammetry

Table S2 Comparison of Spatiotemporal Resolution in Multimodal Electrode Systems

| **Electrode** | **Electrode size** | **Impedance**  **(kΩ at 1kHz)** | **Electrophysiological temporal resolution (μs)** | **Electrochemical temporal resolution (ms)** | **LOD (nM)** | **Matrix** | **Reference** |
| --- | --- | --- | --- | --- | --- | --- | --- |
| GCF-MEAs | ~800 μm² | >10 | 100 | 100 | 50 | *In vivo* | ^[1]^ |
| PEDOT/CNT-MEAs | Φ 35 μm | 74.79 ± 23.01 | 40 | 40 | 50 | *In vivo* | ^[2]^ |
| PEDOT/CNT-MEAs | Φ 37 μm | 3.8 ± 0.4 | - | 40 | 100 | *In vivo* | ^[7]^ |
| Pt/Ir-MEAs | 450 μm² | 579 ± 22 | 40 | 20 | 100 | *In vivo* | ^[5]^ |
| MEAs | Φ 30 μm | 500–800 | 50 | - | - | *In vivo* | ^[8]^ |
| Pt/GlOx-BSA-GDH/mPD | 7500 µm² | - | - | 100 | 160 | *In vitro* | ^[9]^ |
| CFM/Pt/Nafion | - | - | - | 1000 | 50 | *In vivo* | ^[6]^ |
| This work | Φ 20 μm | 1.48 ± 0.61 | 33 | 20 | 25 | *In vivo* |  |

* LOD—Lower limit of detection; GCF—Glassy carbon fiber-like; MEAs—Microelectrode arrays; PEDOT—Poly (3,4-ethylene dioxythiophene); CNT—Carbon nanotube; Pt—Platinum; Ir—Iridium; GlOx—Glutamate oxidase; BSA—Bovine serum albumin; GDH—Glutaraldehyde; mPD—m-Phenylenediamine; CFM—Carbon fiber microelectrode


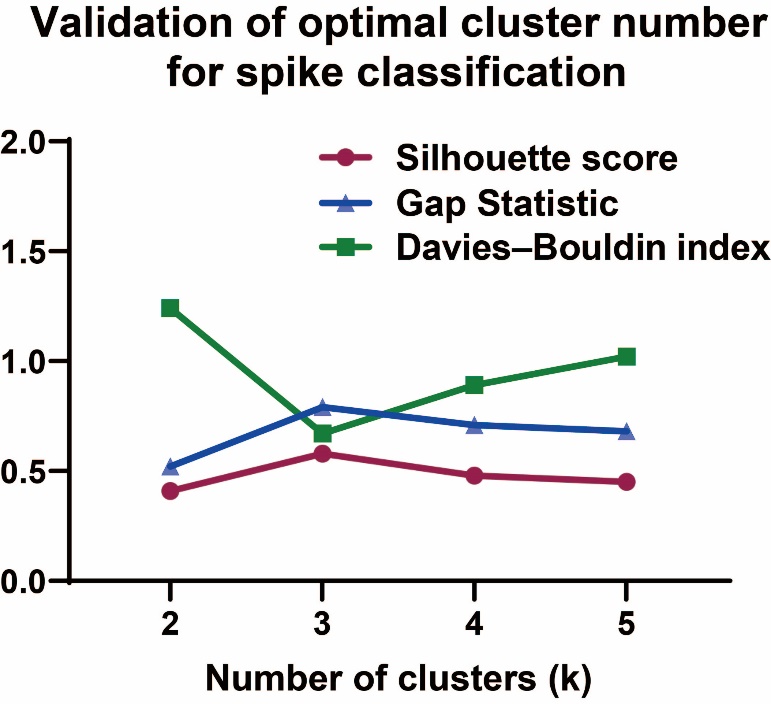


**Supplementary Figure S10.** Silhouette score, gap statistic, and Davies–Bouldin index for determining the optimal number of neuronal clusters.

**Synchronization of neural, dopamine, and behavioral data**

Electrophysiological signals (spikes and local field potentials, LFPs) and polysomnographic signals (EEG and EMG) were recorded simultaneously on a multichannel system (Blackrock Microsystems), ensuring microsecond-scale temporal precision. Electrochemical dopamine signals were recorded on a separate workstation. A software-based timestamp alignment was employed to synchronize dopamine data with neural and behavioral data. At the start and end of each session, the setup was gently tapped to generate simultaneous artifacts in both systems, with additional taps every 3 h during 12-h recordings to monitor clock drift. Custom scripts identified tap timestamps to calculate baseline offsets and correct linear clock drift across the session. Sleep–wake states (wake, NREM, REM) were scored offline in 5-s epochs based on synchronized EEG/EMG signals, and these state labels, along with transient events, were programmatically mapped onto the aligned dopamine and neural signals. This approach ensured accurate correlation of neurochemical and electrophysiological dynamics with sleep-wake transitions, including short-duration events.

REFERENCES

[1] Castagnola E, Cao Q, Robbins E, Wu B, Pwint M Y, Siwakoti U, Cui X T 2025 *Adv. Mater. Technol.* **10** 2400863

[2] Wu B, Castagnola E, McClung C A, Cui X T 2024 *Advanced Science* **11** 2308212

[3] Hasegawa A, Matsuda N, Suzuki I 2025 *Biosens. Bioelectron.* **287** 117696

[4] Xiao G, Song Y, Zhang Y, Xing Y, Zhao H, Xie J, Xu S, Gao F, Wang M, Xing G, Cai X 2019 *ACS Sens.* **4** 1992

[5] Johnson M D, Franklin R K, Gibson M D, Brown R B, Kipke D R 2008 *J. Neurosci. Methods* **174** 62

[6] Fernandes E, Ledo A, Gerhardt G A, Barbosa R M 2024 *Talanta* **268** 125302

[7] Taylor I M, Patel N A, Freedman N C, Castagnola E, Cui X T 2019 *Anal. Chem.* **91** 12917

[8] Mannal N, Kleiner K, Fauler M, Dougalis A, Poetschke C, Liss B 2021 *Front. Synaptic Neurosci.* **13**

[9] Scoggin J L, Tan C, Nguyen N H, Kansakar U, Madadi M, Siddiqui S, Arumugam P U, DeCoster M A, Murray T A 2019 *Biosens. Bioelectron.* **126** 751
