# Supplementary figures and images for: Targeted-Modified MultiTransm Microelectrode Arrays Simultaneously Track Dopamine and Cellular Electrophysiology in Nucleus Accumbens during Sleep–Wake Transitions
Source: Research (Wash D C). 2025 Oct 9;8:0944. doi: 10.34133/research.0944 (PMC12509213; doi:10.34133/research.0944)

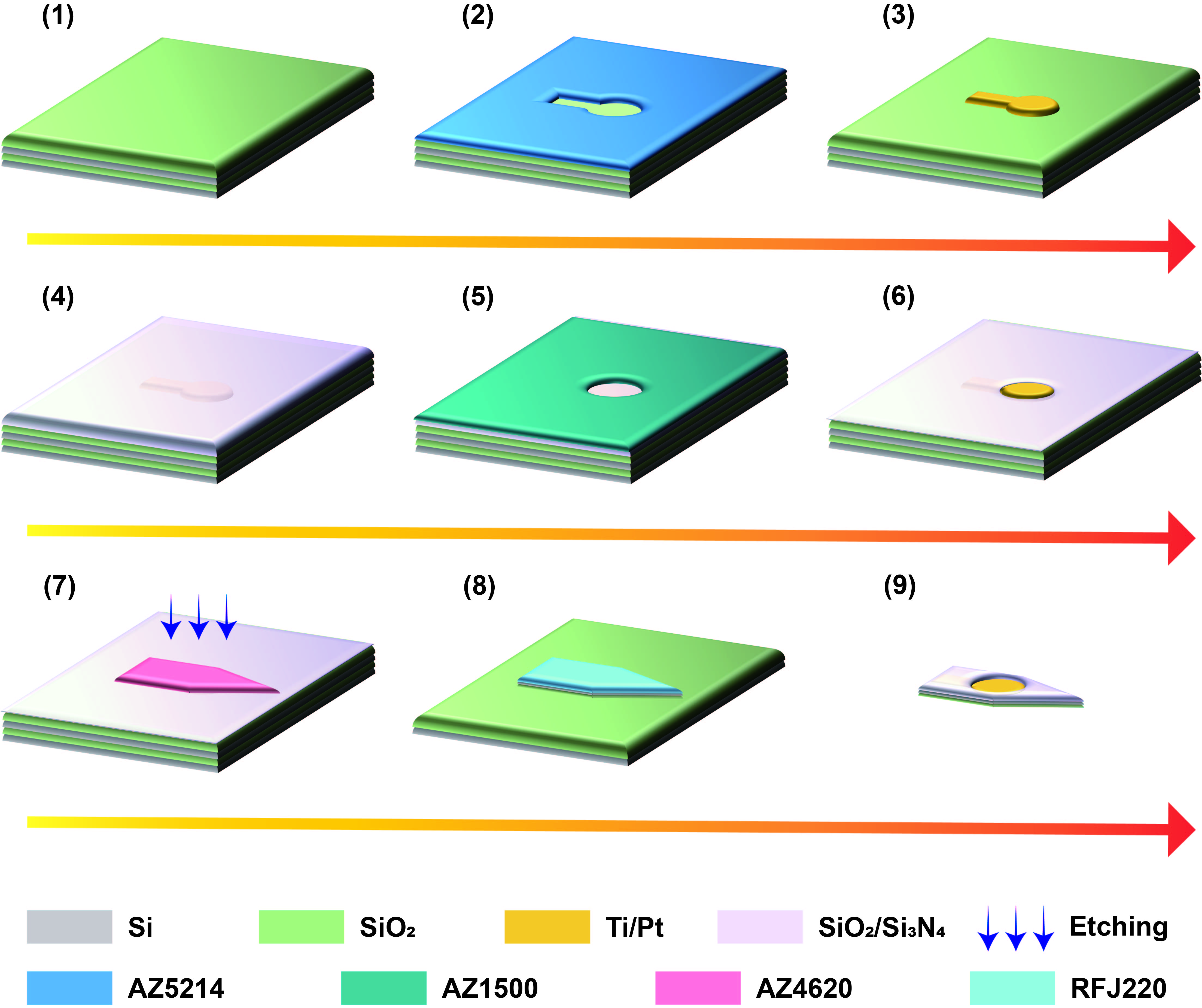

Supplement: Supplementary 1 — Figs. S1 to S10 Tables S1 and S2 [file research.0944.f1.zip › Figure S1.jpg]

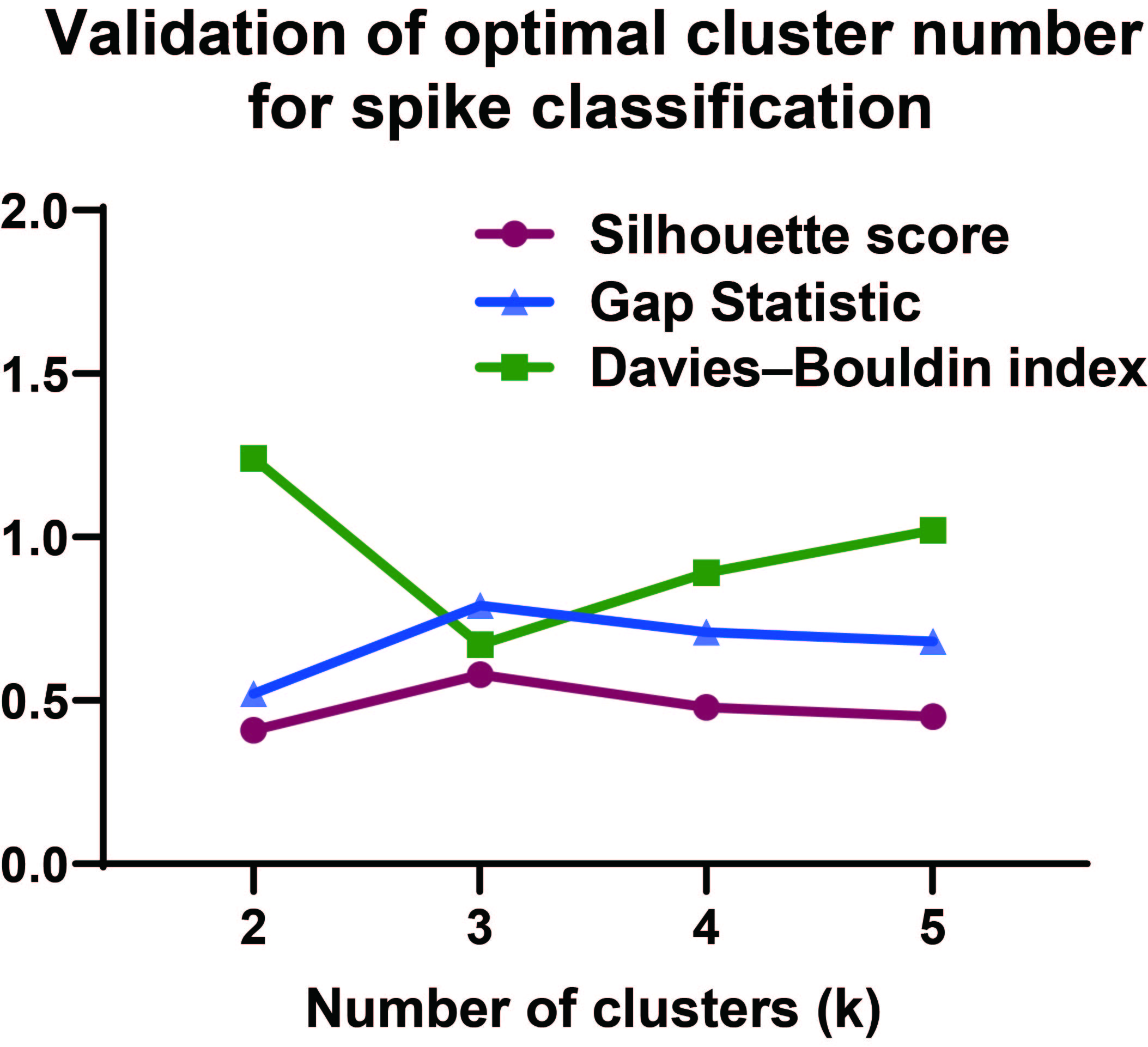

Supplement: Supplementary 1 — Figs. S1 to S10 Tables S1 and S2 [file research.0944.f1.zip › Figure S10.jpg]

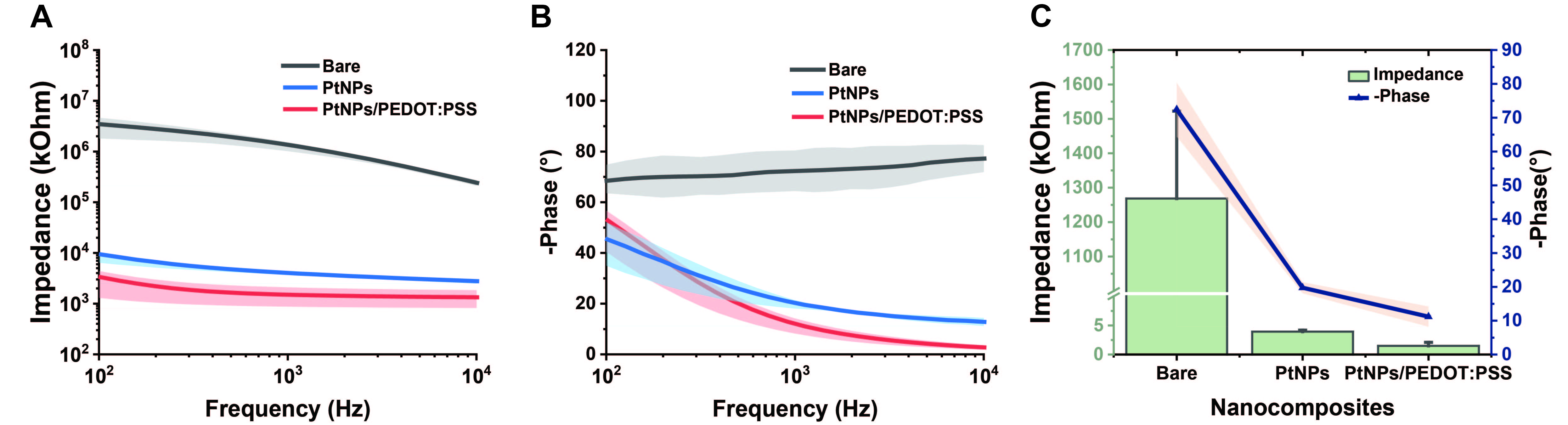

Supplement: Supplementary 1 — Figs. S1 to S10 Tables S1 and S2 [file research.0944.f1.zip › Figure S2.jpg]

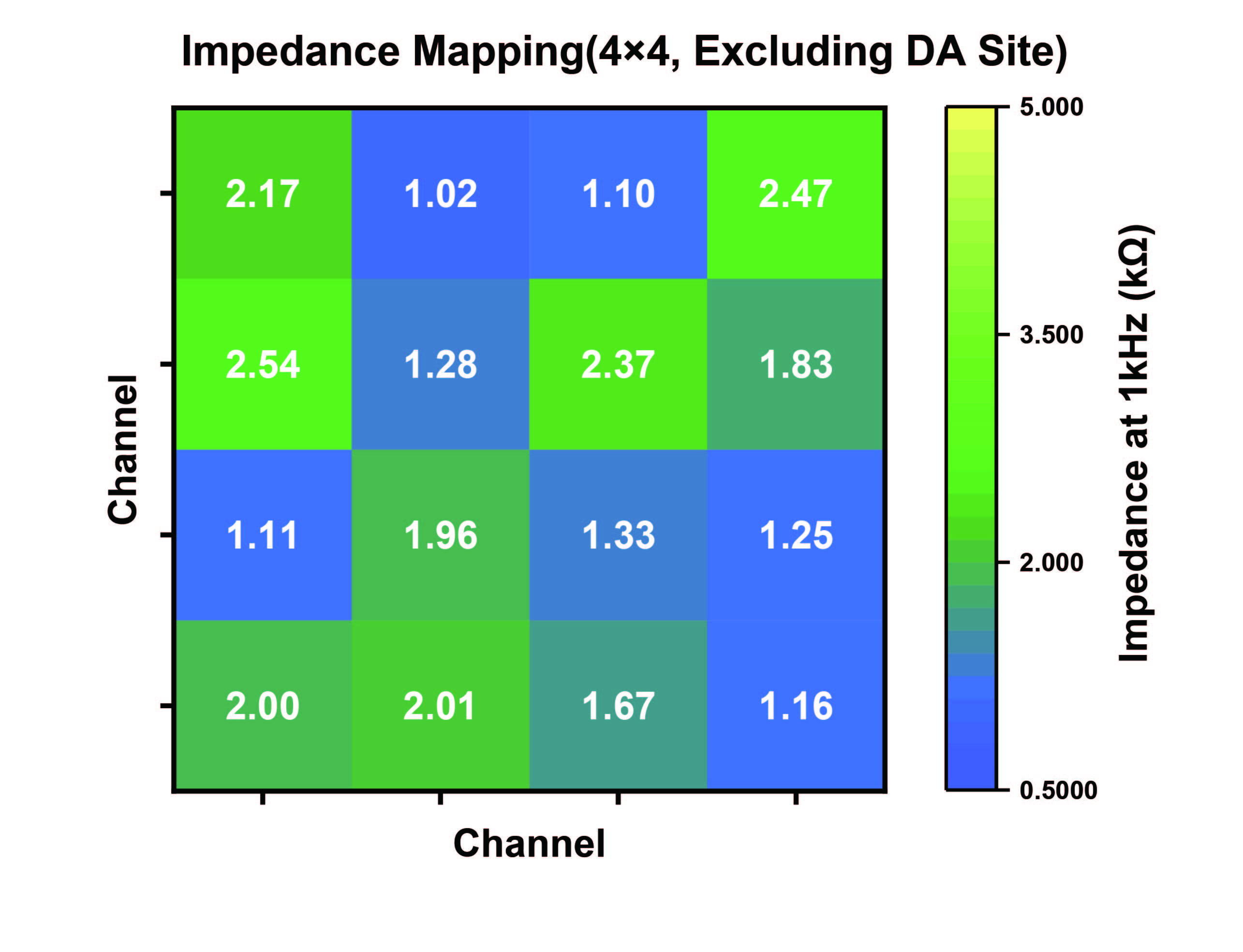

Supplement: Supplementary 1 — Figs. S1 to S10 Tables S1 and S2 [file research.0944.f1.zip › Figure S3.jpg]

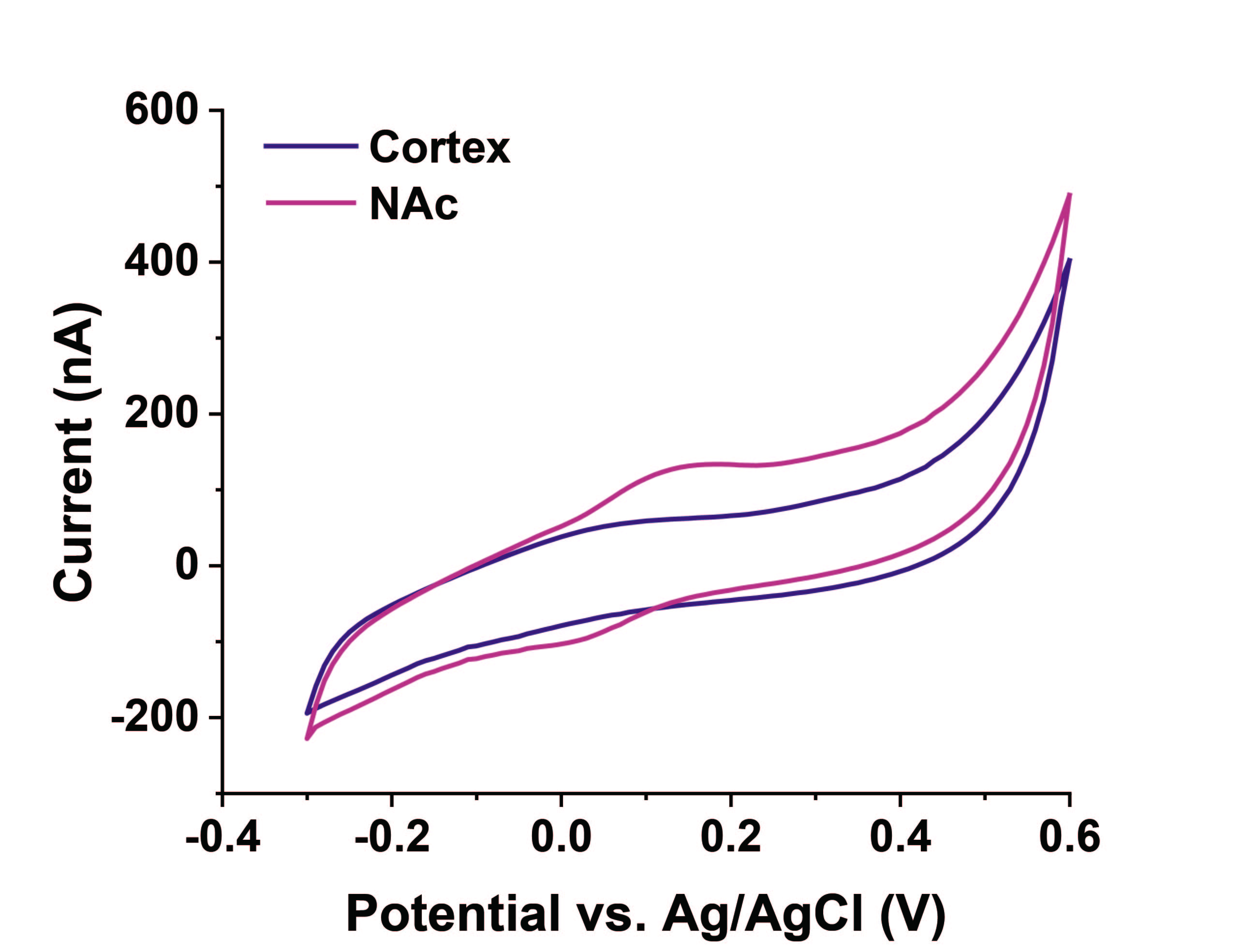

Supplement: Supplementary 1 — Figs. S1 to S10 Tables S1 and S2 [file research.0944.f1.zip › Figure S4.jpg]

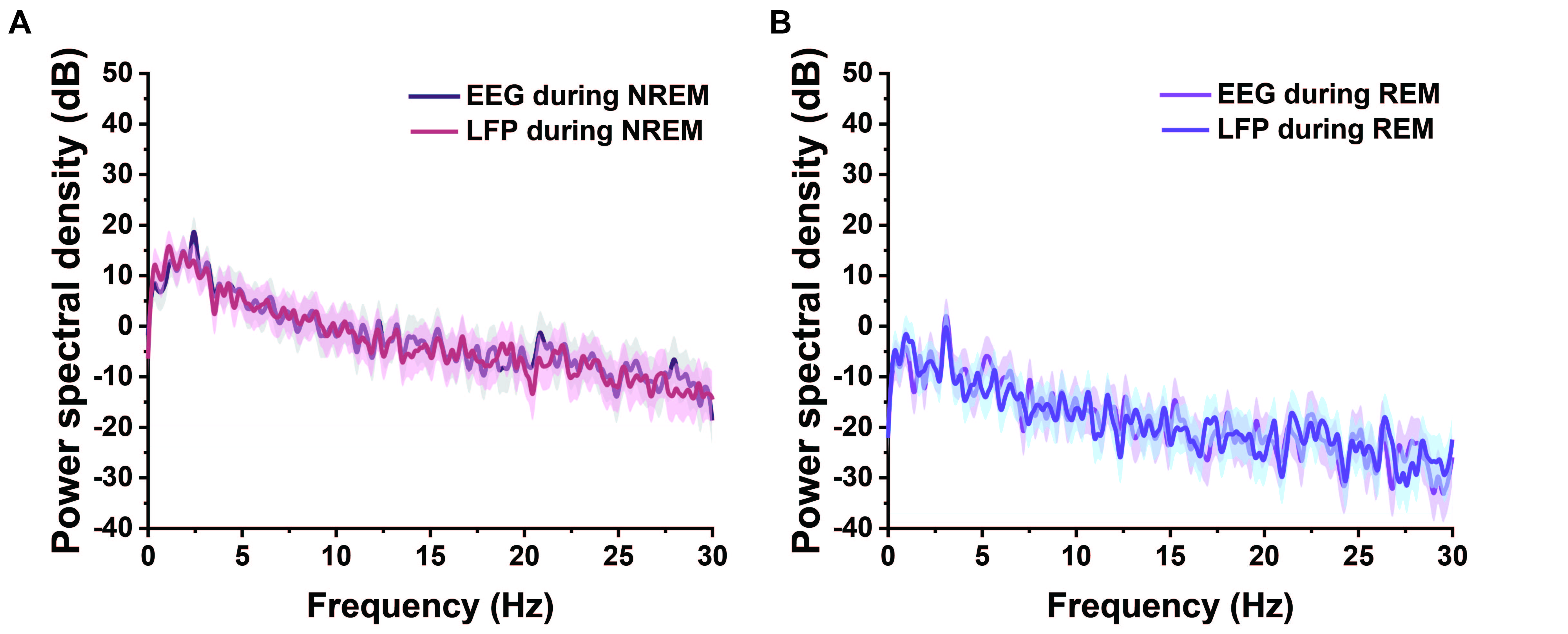

Supplement: Supplementary 1 — Figs. S1 to S10 Tables S1 and S2 [file research.0944.f1.zip › Figure S5.jpg]

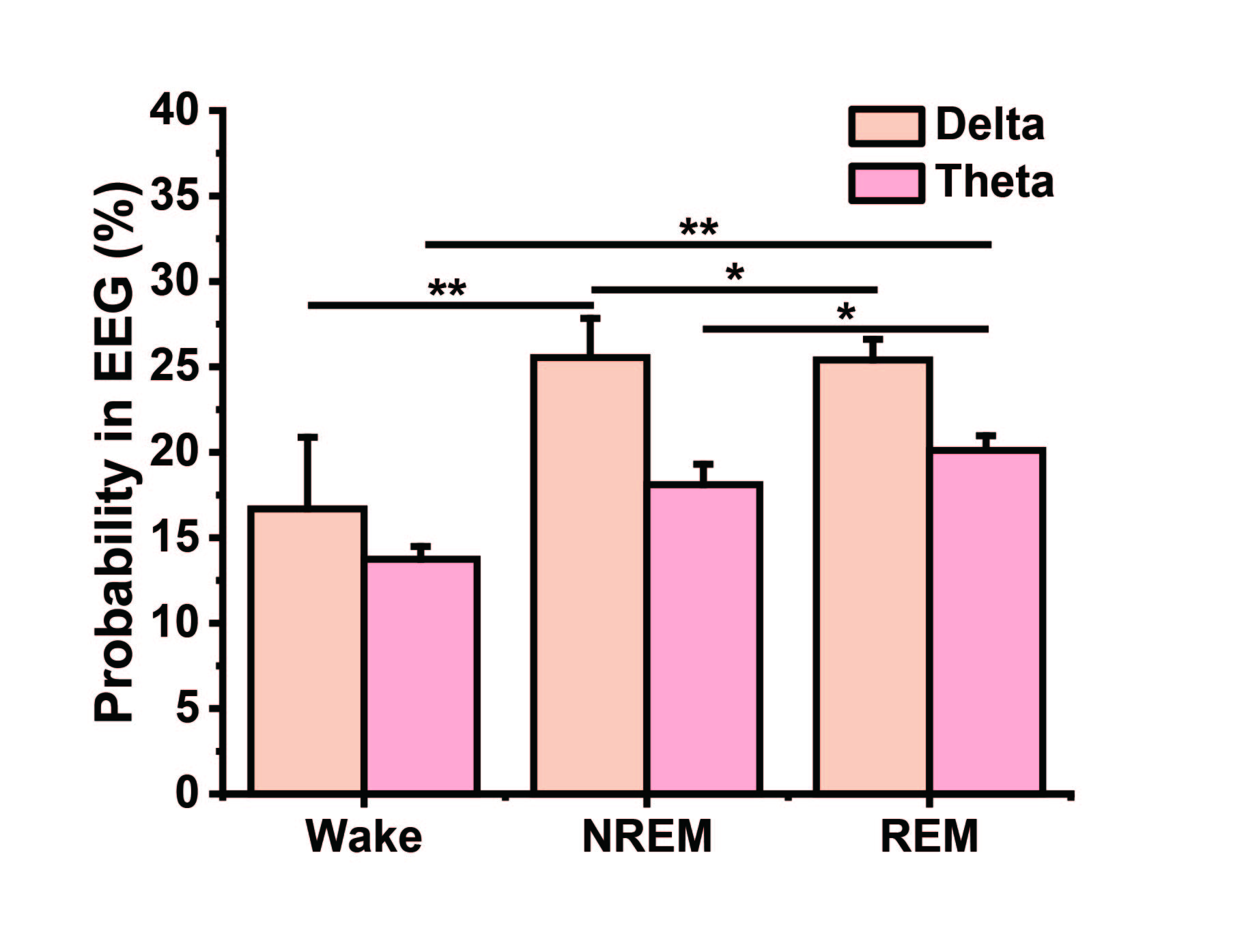

Supplement: Supplementary 1 — Figs. S1 to S10 Tables S1 and S2 [file research.0944.f1.zip › Figure S6.jpg]

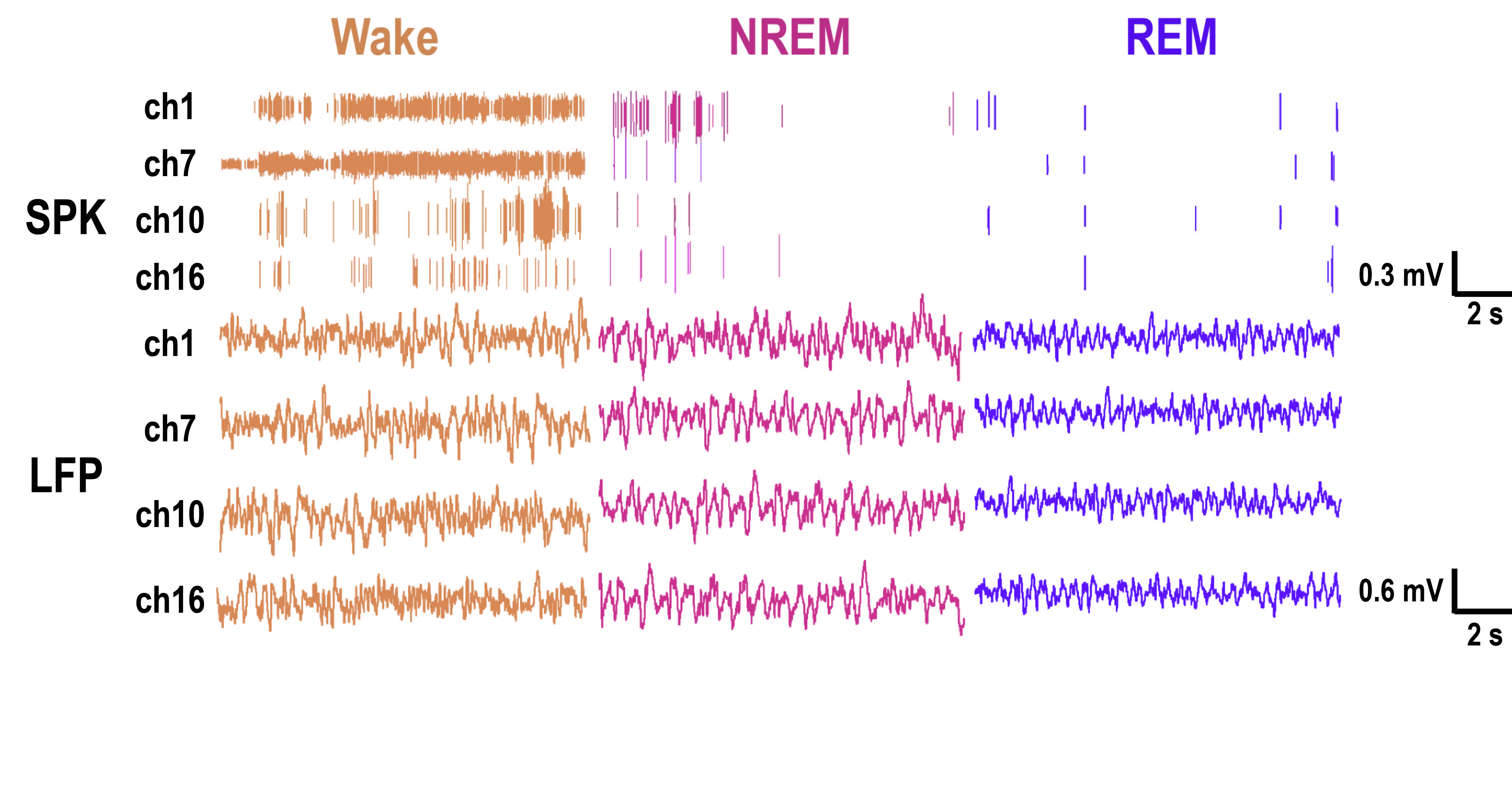

Supplement: Supplementary 1 — Figs. S1 to S10 Tables S1 and S2 [file research.0944.f1.zip › Figure S7.jpg]

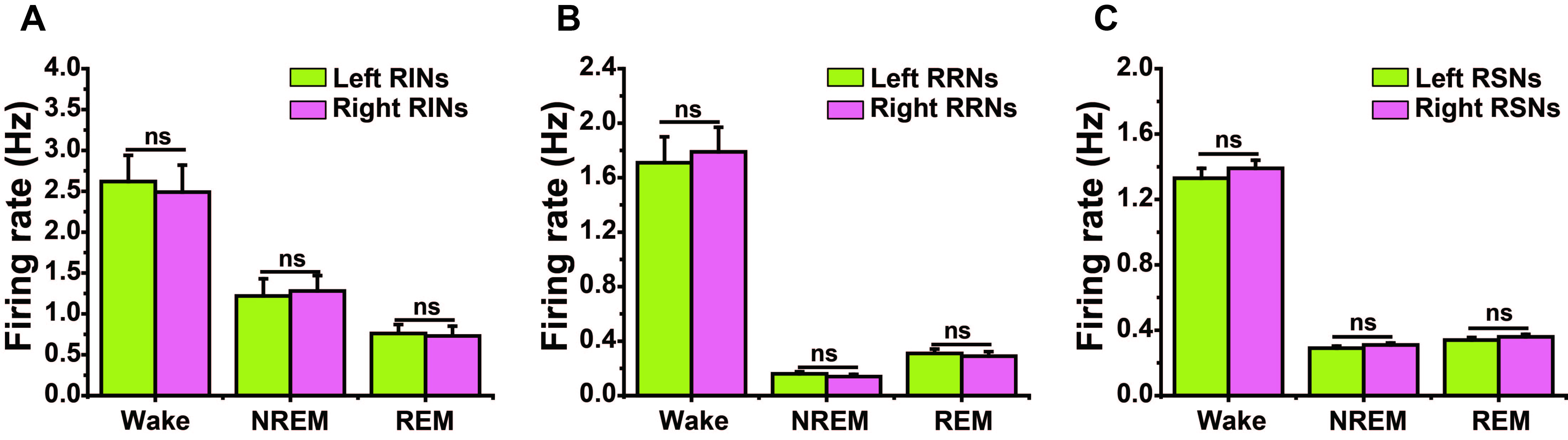

Supplement: Supplementary 1 — Figs. S1 to S10 Tables S1 and S2 [file research.0944.f1.zip › Figure S8.jpg]

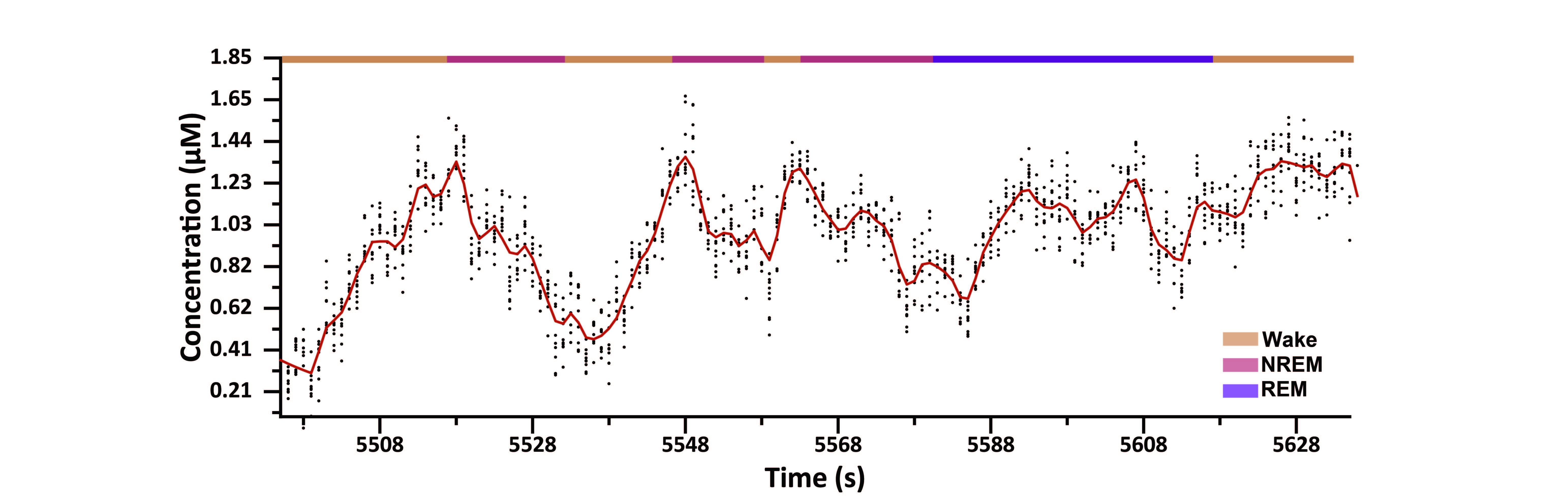

Supplement: Supplementary 1 — Figs. S1 to S10 Tables S1 and S2 [file research.0944.f1.zip › Figure S9.jpg]
